# Biogeographic history and cryptic diversity of saxicolous Tropiduridae lizards endemic to the semiarid Caatinga

**DOI:** 10.1186/s12862-015-0368-3

**Published:** 2015-05-23

**Authors:** Fernanda P Werneck, Rafael N Leite, Silvia R Geurgas, Miguel T Rodrigues

**Affiliations:** Programa de Coleções Científicas Biológicas, Instituto Nacional de Pesquisas da Amazônia, Av. André Araújo 2936, 69060-000 Manaus, AM Brazil; Departamento de Zoologia, Universidade de Brasília, 70910-900 Brasília, DF Brazil; Coordenação de Biodiversidade, Instituto Nacional de Pesquisas da Amazônia, Av. André Araújo 2936, 69060-000 Manaus, AM Brazil; Departamento de Zoologia, Instituto de Biociências, Universidade de São Paulo, 05508-090 São Paulo, SP Brazil

**Keywords:** Neotropics, Northeastern Brazil, Speciation, Species delimitation, Bayesian phylogeography, *Tropidurus semitaeniatus* species group, Squamata

## Abstract

**Background:**

Phylogeographic research has advanced in South America, with increasing efforts on taxa from the dry diagonal biomes. However, the diversification of endemic fauna from the semiarid Caatinga biome in northeastern Brazil is still poorly known. Here we targeted saxicolous lizards of the *Tropidurus semitaeniatus* species group to better understand the evolutionary history of these endemic taxa and the Caatinga. We estimated a time-calibrated phylogeny for the species group based on two mitochondrial and two nuclear genes and jointly estimated the species limits and species tree within the group. We also devoted a denser phylogeographic sampling of the *T. semitaeniatus* complex to explore migration patterns, and the spatiotemporal diffusion history to verify a possible role of the São Francisco River as a promoter of differentiation in this saxicolous group of lizards.

**Results:**

Phylogenetic analysis detected high cryptic genetic diversity, occurrence of unique microendemic lineages associated with older highlands, and a speciation history that took place during the Pliocene-Pleistocene transition. Species delimitation detected five evolutionary entities within the *T. semitaeniatus* species group, albeit with low support. Thus, additional data are needed for a more accurate definition of species limits and interspecific relationships within this group. Spatiotemporal analyses reconstructed the geographic origin of the *T. semitaeniatus* species complex to be located north of the present-day course of the São Francisco River, followed by dispersal that expanded its distribution towards the northwest and south. Gene flow estimates showed higher migration rates into the lineages located north of the São Francisco River.

**Conclusions:**

The phylogenetic and population structures are intrinsically associated with stable rock surfaces and landscape rearrangements, such as the establishment of drainage basins located to the northern and southern distribution ranges. The *T. semitaeniatus* complex preserved high genetic diversity during range expansion, possibly as a result of frequent long-distance dispersal events. Our results indicate that both the current course of the São Francisco River and its paleo-courses had an important role in promoting diversification of the Caatinga endemic *T. semitaeniatus* species group.

**Electronic supplementary material:**

The online version of this article (doi:10.1186/s12862-015-0368-3) contains supplementary material, which is available to authorized users.

## Background

The South American continent is reputed for its remarkable biodiversity and intricate evolutionary history [1]. A renewed interest in understanding the biotic diversification of South America has emerged in the past decade [1] thanks to advances in the field of biogeography driven by the integrative role of molecular genetics [2]. Although this continent has been overlooked in terms of phylogeographic studies for virtually all taxonomic groups [3], a number of recent molecular investigations shed new light on the patterns and processes governing the historical evolution of the South American herpetofauna. In common, they point to the prevalence of cryptic diversity across several amphibian and reptilian taxa [4-10].

**Figure 1:**
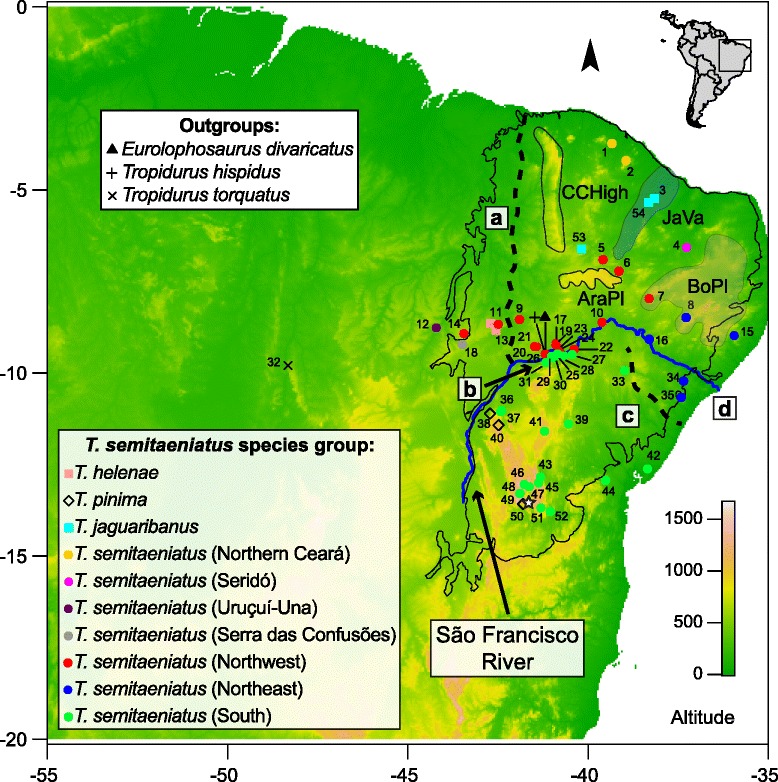
Localities sampled for the *Tropidurus semitaeniatus* species group and outgroups. Limits of the Caatinga are outlined in black and the current São Francisco River configuration in blue. Numbers correspond to locality names in Table 1 and colored symbols follow the same colors for clades in Figure 2. The white star right by locality number 50 is the type locality for *T. semitaeniatus* (Sincorá Velho, BA). The major geomorphological features discussed in the text are highlighted (AraPl = Araripe Plateau; CCHigh = Central Ceará Highlands/Serra da Ibiapaba, JaVa = Jaguaribe river valley, BoPl = Borborema Plateau). Major paleocourse phases of the SFR correspond to: **(a)** early opening to the equatorial Atlantic Ocean; **(b)** paleolacustrine phase; **(c)** forsaken meanders located to the south of the present mouth; and **(d)** the current course.

However, most of the studies originally addressing Neotropical diversification have focused on taxa restricted to rainforests. Only more recently, investigators also aimed to understand biogeographic patterns of species distributed across the wide-ranging corridor of open-dry habitats that separates the two largest forested biomes in South America, the Atlantic and Amazon rainforests [5,9,11-15]. The open-dry formations, often collectively referred to as the dry diagonal biomes, extend in a NE–SW direction from the semiarid Caatinga of northeastern Brazil to the dry Chaco in Argentina, Bolivia and Paraguay, across the Cerrado savanna of central Brazil (Figure 1 at [11]). While recent investigations have focused on the diversification of herpetofauna groups that are either endemic or typical of the Cerrado [12,16], or broadly distributed across all biomes [5,9,17], no study to date addressed lizard taxa endemic to the biomes located at the extremes of the dry diagonal, namely the Chaco and Caatinga. The latter constitutes the largest nuclei of Seasonally Dry Tropical Forests (SDTF) in South America.

The northeastern edge of the dry diagonal comprises the Brazilian Caatinga, encompassing an area of about 850.000 km^2^ characterized by deciduous xerophytic and thorny vegetation where cactus, shrubs and small trees are abundant. Mean annual temperatures vary between 27–29°C and precipitation is sporadic, ranging from 300–800 mm. The rocky terrain of carved relief comprises large isolated crystalline plateaus reaching up to 1000 m, such as Serra da Borborema, Chapada do Araripe, Serra do Ibiapaba, and Chapada Diamantina, overlooking lowland pediplains strewn with residual massifs and inselbergs (or Monadnocks) [18-20]. The present Caatinga landscape, which is dominated by Precambrian rocks of the São Francisco Craton, was set in place during the Cretaceous and post-Cretaceous denudation that resulted in the establishment of old erosion steeped surfaces, whereas pediplains (or depressions) were formed more recently in the Neogene [18,19,21]. Areas geomorphologically more stable withstood erosion much longer as residual landforms with disparate environmental conditions from those found in the pediplains [20,22], which can act as barriers to gene flow for taxa associated with such residual habitats, thus creating a sky-island setting [23-25]. Because plateaus and inselbergs were formed mostly by erosion rather than uplift, their geological surfaces are typically older than the adjacent pediplains. However, recent investigations pointed that low surfaces in the Caatinga are not systematically younger than the upper ones due to regional episodes of topographic inversion [20,26].

Drainage is mostly intermittent due to the semiarid climate, except for the Parnaíba and São Francisco rivers, with the latter being the major perennial watercourse that intersects a large extent of the Caatinga. The São Francisco River (SFR) has served historically as an important *cis*-Andean center of diversification [27]. For instance, Quaternary sand dunes in the middle SFR show high endemism levels associated with vicariant sister species [28-30] and geographically structured populations [9] from opposite margins which highlight its role in promoting phylogeographic structure and species differentiation. Considering the size of its watershed and historical relevance for the biotic diversification of the Caatinga, the origin and geomorphological evolution of the SFR deserve far more attention than it has received [31]. The SFR is born in Serra da Canastra, state of Minas Gerais, and thence runs northwardly until its course turns abruptly towards the Atlantic Ocean, marking the borderlines of Bahia, Pernambuco, Alagoas and Sergipe states. Nevertheless, geomorphological evidences indicate that the SFR’s paleocourse differed from its present configuration considerably, with distinct paleo-drainage phases determined by inland tectonics and climate change. An early opening possibly connected the paleo-SFR to the equatorial Atlantic Ocean northwest of the present-day Piauí and Parnaíba rivers that could have persisted until the Middle–Late Miocene (Figure 1a;[31,32]. The SFR paleocourse was then interrupted due to uplift of regional sierras and, as proposed by the paleolacustrine hypothesis, became endorrheic carrying Quaternary sand deposits to lacustrine or palustrine depressions of the Remanso–Petrolina area in the middle SFR [27,33-35]. Water level fluctuations during the Late Pleistocene climatic fluctuations exposed these sand deposits previously accumulated in the middle SFR, uncovering wind-activated relict sand dunes (Figure 1b; [27,36]. In a subsequent period, supposedly during the Mindel glaciation, ca. 450 ka, the paleo-SFR found its way out to the Atlantic Ocean through forsaken meanders located south of the present mouth (Figure 1c), which may have acted as diversification barriers, until the SFR reached its current configuration (Figure 1d; [31,33]. In addition to historical changes in the SFR’s courses, the semiarid Caatinga also experienced major geomorphological restructuring due to neotectonics adjustments and intense erosional processes during glaciation times [22].

The geological history of the Brazilian Caatinga is complex and still a matter of debate [11]. Nevertheless, the historical evolution of animal groups adapted to this semiarid biome can help understanding possible mechanisms that account for the biotic diversification in the region and thus its formation history. For example, is there a role for the SFR in the differentiation of the Caatinga’s biota? Are there unique lineages associated with supposedly older highlands? Are there any instances of microendemism within the Caatinga?

The saxicolous tropidurid lizards of the *Tropidurus semitaeniatus* species group [37] are an ideal target to address such biogeographic questions. This monophyletic group comprises four species with marked ecomorphological adaptations to a saxicolous life history, such as prominent dorsoventral body flattening, cryptic coloration, fixed clutch size with two elongated eggs, and strongly keeled tarsal scales that can reduce capture by predators. *Tropidurus semitaeniatus* was described by Spix in 1825 from Serra do Sincorá, state of Bahia, occurring across all the Caatinga biome and eventually at isolated rock outcrops in the vicinities of the Cerrado and Atlantic Forest biomes. Although slight geographical variation in color patterns has been documented for *T. semitaeniatus* [28], the taxon was never properly investigated for the existence of cryptic species and the evolutionary basis of such color variation. The three other congeners have more restricted allopatric distributions. *Tropidurus pinima* (Rodrigues, 1984) occurs only in the surroundings of Serra do Assuruá, in northern Bahia, *T. helenae* (Manzani & Abe, 1990) inhabits the area of the Parque Nacional da Serra da Capivara, state of Piauí [38-40], and the recently described *T. jaguaribanus* Passos, Lima & Borges-Nojosa, 2011 is from the Jaguaribe valley, in eastern Ceará state [41,42]. Despite some marginal occurrence of *T. semitaeniatus* near transitional areas [43], the species group is distributed throughout the Caatinga and can be considered endemic to this semiarid biome [37,38]. *Tropidurus semitaeniatus* is dependent on rock outcrops where the lizards seek shelter in crevices, which is an example of ecomorphological convergence with its African counterpart, the cordylid genus *Platysaurus*. This extreme site specificity reduces dispersal opportunities across non-rocky environments interspersed in the landscape, favoring the idea that the evolutionary history of the group should reflect key geomorphological events in the region.

Herein, we investigate the historical biogeography of the saxicolous *Tropidurus semitaeniatus* species group using a molecular approach to better understand the biotic diversification associated with rocky formations of the semiarid Caatinga. We employ DNA sequence data from mitochondrial and nuclear markers to estimate a time-calibrated species tree for all recognized species within this lizard group. In addition, we address patterns of cryptic diversity and microendemism using species delimitation methods, with taxonomic and conservation implications. Finally, we explore the phylogeography of the broadly distributed *T. semitaeniatus* species complex and the role of the SFR as a barrier by applying Bayesian methods to estimate continuous-space dispersal and migration rates.

## Methods

### Sampling and data collection

We sequenced a total of 138 individuals including all four species of the *T. semitaeniatus* species group for two mitochondrial (the ribosomal RNA, 16S and the cytochrome-b, Cytb) and two protein-coding nuclear genes (the brain-derived neurotrophic factor, BDNF and the phosducin, PDC; Table 1; Figure 1), plus three outgroup taxa: *Eurolophosaurus divaricatus* (Rodrigues, 1986), *T. hispidus* (Spix, 1825), and *T. torquatus* (Wied, 1820). The nuclear DNA (nuDNA) sequence data are a subset of the mitochondrial DNA (mtDNA) data set. We deposited all sequences in GenBank (accession numbers: KR706819-KR707320).

**Table 1:** **Details of material examined from the** ***Tropidurus semitaeniatus*** **species group and outgroups, with locality data mtDNA haploclades assignments**

**Species**	**Voucher**	**Locality (Codes)**	**Lat**	**Long**	**mtDNA haploclade**
*Eurolophosaurus divaricatus* (OG)	MTR11746	Alagoado, BA (26)	−9.486	−41.189	Ediv
*T. hispidus* (OG)	MTR11239	Alagoado, BA (26)	−9.486	−41.189	This
*T. torquatus* (OG)	MTR8744	Lajeado, TO (32)	−9.795	−48.326	Ttorq
*T. helenae*	S/N	Serra da Capivara, PI (13)	−8.832	−42.553	Thel
*T. helenae*	MTR21452	Serra da Capivara, PI (13)	−8.832	−42.553	Thel
*T. helenae*	MTR23458	PARNA Serra da Capivara, PI (11)	−8.649	−42.702	Thel
*T. helenae*	MTR23481	PARNA Serra da Capivara, PI (11)	−8.649	−42.702	Thel
*T. helenae*	MTR23484	PARNA Serra da Capivara, PI (11)	−8.649	−42.702	Thel
*T. jaguaribanus*	3650	Tabuleiro do Norte, CE (3)	−5.242	−38.160	Tjagua
*T. jaguaribanus*	TEC2242	Açude Benguê, Aiuaba, CE (53)	−6.601	−40.145	Tjagua
*T. jaguaribanus*	TEC2245	Açude Benguê, Aiuaba, CE (53)	−6.601	−40.145	Tjagua
*T. jaguaribanus*	TEC2246	Açude Benguê, Aiuaba, CE (53)	−6.601	−40.145	Tjagua
*T. jaguaribanus*	TEC2292	São João do Jaguaribe, CE (54)	−5.274	−38.263	Tjagua
*T. jaguaribanus*	TEC2295	São João do Jaguaribe, CE (54)	−5.274	−38.263	Tjagua
*T. jaguaribanus*	TEC2296	São João do Jaguaribe, CE (54)	−5.274	−38.263	Tjagua
*T. jaguaribanus*	TEC2300	Açude Benguê, Aiuaba, CE (53)	−6.601	−40.145	Tjagua
*T. jaguaribanus*	TEC2301	Açude Benguê, Aiuaba, CE (53)	−6.601	−40.145	Tjagua
*T. jaguaribanus*	TEC2302	Açude Benguê, Aiuaba, CE (53)	−6.601	−40.145	Tjagua
*T. jaguaribanus*	TEC2303	Açude Benguê, Aiuaba, CE (53)	−6.601	−40.145	Tjagua
*T. jaguaribanus*	TEC2304	Açude Benguê, Aiuaba, CE (53)	−6.601	−40.145	Tjagua
*T. jaguaribanus*	TEC2305	Açude Benguê, Aiuaba, CE (53)	−6.601	−40.145	Tjagua
*T. jaguaribanus*	TEC2306	Açude Benguê, Aiuaba, CE (53)	−6.601	−40.145	Tjagua
*T. jaguaribanus*	TEC2307	Açude Benguê, Aiuaba, CE (53)	−6.601	−40.145	Tjagua
*T. pinima*	91.6574	Rio de Contas, BA (50)	−13.579	−41.802	Tpini
*T. pinima*	91.6575	Rio de Contas, BA (30)	−13.579	−41.802	Tpini
*T. pinima*	MTR2997	Santo Inácio, BA (38)	−11.110	−42.722	Tpini
*T. pinima*	MTR2999	Santo Inácio, BA (38)	−11.110	−42.722	Tpini
*T. pinima*	MTR3004	Santo Inácio, BA (38)	−11.110	−42.722	Tpini
*T. pinima*	MTR3256	Gentio do Ouro, BA (40)	−11.426	−42.482	Tpini
*T. pinima*	MTR3258	Gentio do Ouro, BA (40)	−11.426	−42.482	Tpini
*T. semitaeniatus*	221	Ibateguara, AL (15)	−8.980	−35.947	NE
*T. semitaeniatus*	222	Ibateguara, AL (15)	−8.980	−35.947	NE
*T. semitaeniatus*	223	Ibateguara, AL (15)	−8.980	−35.947	NE
*T. semitaeniatus*	224	Ibateguara, AL (15)	−8.980	−35.947	NE
*T. semitaeniatus*	225	Ibateguara, AL (15)	−8.980	−35.947	NE
*T. semitaeniatus*	226	Ibateguara, AL (15)	−8.980	−35.947	NE
*T. semitaeniatus*	227	Ibateguara, AL	−8.980	−35.947	NE
*T. semitaeniatus*	228	Ibateguara, AL (15)	−8.980	−35.947	NE
*T. semitaeniatus*	229	Ibateguara, AL (15)	−8.980	−35.947	NE
*T. semitaeniatus*	876554	Mucugê, BA (45)	−13.008	−41.372	S
*T. semitaeniatus*	91.6246	Tabatinga, BA (36)	−11.021	−42.398	S
*T. semitaeniatus*	CGERVO096	Capitão Gervásio Oliveira, PI (9)	−8.539	−41.904	NW
*T. semitaeniatus*	CGERVO097	Capitão Gervásio Oliveira, PI (9)	−8.539	−41.904	NW
*T. semitaeniatus*	MTR11697	Sobradinho, BA (23)	−9.386	−40.802	NW
*T. semitaeniatus*	MTR11698	Sobradinho, BA (23)	−9.386	−40.802	NW
*T. semitaeniatus*	MTR11699	Sobradinho, BA (23)	−9.386	−40.802	NW
*T. semitaeniatus*	MTR11703	Serra da Pimenta, BA (19)	−9.386	−40.802	NW
*T. semitaeniatus*	MTR11704	Serra da Pimenta, BA (19)	−9.386	−40.802	NW
*T. semitaeniatus*	MTR11705	Serra da Pimenta, BA (19)	−9.386	−40.802	NW
*T. semitaeniatus*	MTR11706	Serra da Pimenta, BA (19)	−9.386	−40.802	NW
*T. semitaeniatus*	MTR11707	Serra da Pimenta, BA (19)	−9.386	−40.802	NW
*T. semitaeniatus*	MTR11708	Serra da Pimenta, BA (19)	−9.386	−40.802	NW
*T. semitaeniatus*	MTR11718	São Gonçalo da Serra, BA (30)	−9.583	−40.948	S
*T. semitaeniatus*	MTR11719	São Gonçalo da Serra, BA (30)	−9.583	−40.948	S
*T. semitaeniatus*	MTR11720	Juazeiro, BA (27)	−9.497	−40.439	S
*T. semitaeniatus*	MTR11721	Juazeiro, BA (27)	−9.497	−40.439	S
*T. semitaeniatus*	MTR11722	Juazeiro, BA (27)	−9.497	−40.439	S
*T. semitaeniatus*	MTR11723	Morro do Cruzeiro, BA (24)	−9.404	−40.811	NW
*T. semitaeniatus*	MTR11725	Serrote do Urubu, PE (22)	−9.361	−40.387	NW
*T. semitaeniatus*	MTR11726	Serrote do Urubu, PE (22)	−9.361	−40.387	NW
*T. semitaeniatus*	MTR11727	Alagoado, BA (26)	−9.486	−41.189	NW
*T. semitaeniatus*	MTR11728	Sítio Serrote, BA (17)	−9.194	−40.890	NW
*T. semitaeniatus*	MTR11729	Sítio Serrote, BA (17)	−9.194	−40.890	NW
*T. semitaeniatus*	MTR11731	Salitre, BA (28)	−9.533	−40.674	S
*T. semitaeniatus*	MTR11733	Salitre, BA (28)	−9.533	−40.674	S
*T. semitaeniatus*	MTR11737	54 km W Casa Nova, BA (21)	−9.283	−41.432	NW
*T. semitaeniatus*	MTR11738	54 km W Casa Nova, BA 21)	−9.283	−41.432	NW
*T. semitaeniatus*	MTR11739	54 km W Casa Nova, BA (21)	−9.283	−41.432	NW
*T. semitaeniatus*	MTR11740	27 km W de Sobradinho, BA (29)	−9.542	−41.016	S
*T. semitaeniatus*	MTR11741	4 km W Pissarão, BA (31)	−9.711	−41.168	S
*T. semitaeniatus*	MTR11742	4 km W Pissarão, BA (31)	−9.711	−41.168	S
*T. semitaeniatus*	MTR11743	4 km W Pissarão, BA (31)	−9.711	−41.168	S
*T. semitaeniatus*	MTR11744	4 km W Pissarão, BA (31)	−9.711	−41.168	S
*T. semitaeniatus*	MTR11752	Serra do Nilo, BA (25)	−9.483	−40.847	S
*T. semitaeniatus*	MTR11753	Serra do Nilo, BA (25)	−9.483	−40.847	S
*T. semitaeniatus*	MTR11754	Serra do Nilo, BA (25)	−9.483	−40.847	S
*T. semitaeniatus*	MTR11759	Serra do Lajedo, BA (20)	−9.280	−41.483	NW
*T. semitaeniatus*	MTR11760	Serra do Lajedo, BA (20)	−9.280	−41.483	NW
*T. semitaeniatus*	MTR11861	Elísio Medrado, BA (44)	−12.936	−39.512	S
*T. semitaeniatus*	MTR11899	Missão Velha, CE (6)	−7.222	−39.143	NW
*T. semitaeniatus*	MTR11900	Missão Velha, CE (6)	−7.222	−39.143	NW
*T. semitaeniatus*	MTR11913	Farias Brito, CE (5)	−6.907	−39.586	NW
*T. semitaeniatus*	MTR11916	Farias Brito, CE (5)	−6.907	−39.586	NW
*T. semitaeniatus*	MTR11918	Farias Brito, CE (5)	−6.907	−39.586	NW
*T. semitaeniatus*	MTR14038	Petrolândia, PE (16)	−9.081	−38.304	NW
*T. semitaeniatus*	MTR14039	Orocó, PE (10)	−8.618	−39.613	NW
*T. semitaeniatus*	MTR15260	N. Sra. da Glória, SE (34)	−10.222	−37.352	NE
*T. semitaeniatus*	MTR15261	N. Sra. da Glória, SE (34)	−10.222	−37.352	NE
*T. semitaeniatus*	MTR15262	N. Sra. da Glória, SE (34)	−10.222	−37.352	NE
*T. semitaeniatus*	MTR15371	Catimbau, PE (8)	−8.487	−37.281	NE
*T. semitaeniatus*	MTR15372	Catimbau, PE (8)	−8.487	−37.281	NE
*T. semitaeniatus*	MTR15373	Catimbau, PE (8)	−8.487	−37.281	NE
*T. semitaeniatus*	MTR15374	Catimbau, PE (8)	−8.487	−37.281	NE
*T. semitaeniatus*	MTR15577	ESEC/Seridó, RN (4)	−6.575	−37.268	Ser
*T. semitaeniatus*	MTR15578	Ibateguara (Mato do Coimbra), AL (15)	−8.980	−35.947	NE
*T. semitaeniatus*	MTR15579	ESEC/Seridó, RN (4)	−6.575	−37.268	Ser
*T. semitaeniatus*	MTR15614	Itaguaçu da Bahia, BA (37)	−11.060	−42.425	S
*T. semitaeniatus*	MTR15615	Itaguaçu da Bahia, BA (37)	−11.060	−42.425	S
*T. semitaeniatus*	MTR15622	Dias D’Ávila, BA (42)	−12.620	−38.354	S
*T. semitaeniatus*	MTR19908	Andaraí, Toca do Morcego, BA (43)	−12.840	−41.322	S
*T. semitaeniatus*	MTR19909	Andaraí, Toca do Morcego, BA (43)	−12.840	−41.322	S
*T. semitaeniatus*	MTR20022	Morro do Chapéu, BA (41)	−11.592	−41.208	S
*T. semitaeniatus*	MTR20025	Mucugê, Serra do Bastião, BA (47)	−13.108	−41.632	S
*T. semitaeniatus*	MTR20074	Catolé de Cima, BA (48)	−13.286	−41.889	S
*T. semitaeniatus*	MTR20075	Catolé de Cima, BA (48)	−13.286	−41.889	S
*T. semitaeniatus*	MTR20079	Pico do Barbado, BA (49)	−13.292	−41.891	S
*T. semitaeniatus*	MTR20129	Serra do Cafundó, BA (46)	−13.045	−41.770	S
*T. semitaeniatus*	MTR20131	Serra do Cafundó, BA (46)	−13.045	−41.770	S
*T. semitaeniatus*	MTR21286	Canudos, Toca Velha, BA (33)	−9.941	−38.987	S
*T. semitaeniatus*	MTR21449	Catimbau, PE (8)	−8.487	−37.281	NE
*T. semitaeniatus*	MTR21450	Catimbau, PE (8)	−8.487	−37.281	NE
*T. semitaeniatus*	MTR21451	Serra Talhada, PE (7)	−7.966	−38.307	NW
*T. semitaeniatus*	MTR22367	Morro do Chapéu, BA (41)	−11.591	−41.207	S
*T. semitaeniatus*	MTR22369	Morro do Chapéu, BA (41)	−11.591	−41.207	S
*T. semitaeniatus*	MTR22942	Barra da Estiva, BA (51)	−13.686	−41.310	S
*T. semitaeniatus*	MTR22990	FLONA Contendas do Sincorá, BA (52)	−13.783	−41.047	S
*T. semitaeniatus*	MTR23494	PARNA Serra da Capivara, PI (11)	−8.673	−42.492	NW
*T. semitaeniatus*	MTR23495	PARNA Serra da Capivara, PI (11)	−8.673	−42.492	NW
*T. semitaeniatus*	MTR23496	PARNA Serra da Capivara, PI (11)	−8.673	−42.492	NW
*T. semitaeniatus*	MTR2576	Uruçuí-Una, PI (12)	−8.768	−44.211	UUna
*T. semitaeniatus*	MTR2577	Uruçuí-Una, PI (12)	−8.768	−44.211	UUna
*T. semitaeniatus*	MTR3752	Alagoado, BA (26)	−9.486	−41.189	NW
*T. semitaeniatus*	MTR3753	Alagoado, BA (26)	−9.486	−41.189	NW
*T. semitaeniatus*	MTR4541	Pacoti, CE (2)	−4.194	−38.951	NoCE
*T. semitaeniatus*	MTR4554	Morrinhos, PI (14)	−8.924	−43.449	NW
*T. semitaeniatus*	MTR4649	Serra das Confusões, PI (18)	−9.219	−43.491	SCon
*T. semitaeniatus*	MTR4650	Serra das Confusões, PI (18)	−9.219	−43.491	SCon
*T. semitaeniatus*	MTR4651	Serra das Confusões, PI (18)	−9.219	−43.491	SCon
*T. semitaeniatus*	RPD056	Miguel Calmon, Parque Estadual Sete Passagens, BA (39)	−11.393	−40.541	S
*T. semitaeniatus*	RPD087	Miguel Calmon, Parque Estadual Sete Passagens, BA (39)	−11.393	−40.541	S
*T. semitaeniatus*	s/n005	Catimbau, PE (8)	−8.487	−37.281	NE
*T. semitaeniatus*	s/n006	Catimbau, PE (8)	−8.487	−37.281	NE
*T. semitaeniatus*	s/n14	Serra Talhada, PE (7)	−7.966	−38.307	NW
*T. semitaeniatus*	UFCL3874	F. Exp. Vale do Curu, Município Pentecoste, CE (1)	−3.730	−39.340	NoCE
*T. semitaeniatus*	UFCL3875	F. Exp. Vale do Curu, Município Pentecoste, CE (1)	−3.730	−39.340	NoCE
*T. semitaeniatus*	UFCL3876	F. Exp. Vale do Curu, Município Pentecoste, CE (1)	−3.730	−39.340	NoCE
*T. semitaeniatus*	UFSEH24	Serra de Itabaiana, SE (35)	−10.667	−37.417	NE
*T. semitaeniatus*	UFSEH25	Serra de Itabaiana, SE (35)	−10.667	−37.417	NE

OG = outgroup species; PARNA = National Park. Brazilian states: BA = Bahia, CE = Ceará, PE = Penambuco, PI = Piauí, TO = Tocantins. mtDNA haploclades: Ediv = *Eurolophosaurus divaricatus* (OG); This = *T. hispidus* (OG); Ttorq = *T. torquatus* (OG); Tpin = *T. pinima;* Thel = T. *helenae;* Tjagua = *T.* jaguaribanus; NW = Northwest, NE = Northeast, S = South, Ser = Seridó, UUna = Uruçuí-Una, NoCE = North Ceará, SCon = Serra Confusões.

We extracted total genomic DNA from liver or muscle tissues using DNeasy Blood & Tissue Kit (QIAGEN), and amplified gene fragments following standard PCR techniques with primers from the literature and developed specifically for this study (Table 2). PCR products were purified with Exonuclease I and Shrimp Alkaline Phosphatase (Thermo Scientific) and directly sequenced on an ABI PRISM 3500 DNA Sequencer (Applied Biosystems) at Instituto de Ciências Biomédicas (Universidade de São Paulo, Brazil) using the Big Dye Terminator v3.1 method according to the manufacturer’s instructions.

**Table 2:** **Primers information used in this study**

**Marker**	**Primers**	**Sequence (5′-3′)**	**Reference**
16S	F	CTGTTTACCAAAAACATMRCCTYTAGC	[114]
	R	TAGATAGAAACCGACCTGGATT	
Cytb	cytTrop	TGAAAAACCAYCGTTATTCAAC	This study
	CB3	GGCGAATAGGAAGTATCATTC	[115]
BDNF	F	GACCATCCTTTTCCTKACTATGGTTATTTCATACTT	[116]
	R	CTATCTTCCCCTTTTAATGGTCAGTGTACAAAC	
PDC	F2	AGATGAGCATGCAGGAGTATGA	[117]
	R1	TCCACATCCACAGCAAAAAACTCCT	

Chromatograms were assembled and edited using the program Geneious v6.1.5 (Biomatters) and multiple sequence alignments were performed with Muscle v3.8.31 [44]. We resolved the gametic phase of heterozygous individuals with PHASE v2.1.1 [45], using default program options: 100 burn-in iterations, 100 main iterations, one thinning interval per iteration, and confidence probability thresholds of 0.90. Models of nucleotide substitution were selected based on the Akaike information criterion as implemented in the program jModelTest v2.1.1 [46].

### Estimation of gene trees, haplotype networks, and summary statistics

We estimated maximum likelihood (ML) gene trees for the concatenated mtDNA data set and for each nuclear loci with phased alleles in the program RAxML v7.2.6 [47]. We implemented the GTR + Γ model with 200 independent ML searches and 1,000 nonparametric bootstrap replicates to assess nodal support. We also estimated a time-calibrated Bayesian tree for the mtDNA loci with BEAST v1.7.5 [48], using five independent runs of 200 million generations each sampled at every 20,000 steps. We checked for stationary posterior distributions, effective sample sizes (ESS) above 200, and convergence between runs by examining parameter traces with the program Tracer v1.6 [49]. We combined runs and trees after removing a 10% burn-in with Log Combiner v1.7.5 [48], and annotated tree files and computed the maximum clade credibility (MCC) tree with TreeAnnotator v1.7.5 [48]. To calibrate divergence times estimates we used a normal prior distribution (mean = 1.94 × 10^−2^ substitutions/million year; SD = 0.346 × 10^−5^) on the global substitution rate of the mtDNA (16S + Cyt *b*) following recent estimates for *Liolaemus* lizards [50], which constitute a genus of the closely-related family Liolaemidae with body sizes comparable to *T. semitaeniatus.* Moreover, such estimates fall within the sequence divergence range of 1.3–2% per million year typically considered for squamate reptiles 1.3-2% per million year [51,52]. We built haplotype networks for visualization of the two nuclear gene (BDNF and PDC) genealogies by converting ML tree estimates with Haploviewer (http://www.cibiv.at/~greg/haploviewer) [53].

We calculated DNA polymorphism statistics to summarize the genetic diversity of each gene and major mtDNA lineages using DnaSP v5.10.1 [54], and calculated the following statistics: number of haplotypes (H), haplotype diversity (Hd), Watterson’s theta (θ_*w*_), nucleotide diversity per site (Pi), average number of nucleotide differences between sequences (k), and number of segregating sites (S). Net among-group distances between major mtDNA haploclades and the outgroup taxa were computed with MEGA v5.2.1 [55] using uncorrected and Tamura-Nei corrected [56] distances, and 500 bootstrap replicates to estimate standard errors.

### Species delimitation

We assessed the evolutionary independence of lineages within the *Tropidurus semitaeniatus* species group by means of a coalescent-based species delimitation approach. We first attempted to validate candidate species using a multilocus Bayesian method implemented in BPP v2.2 [57]. To that purpose, species assignment was either based on mtDNA haploclades or genetic clusters identified by the generalized mixed Yule coalescent (GMYC) model and the species tree topology inferred with *BEAST under each of these configurations was used as guide tree (see below). However, the different models of species delimitation failed to converge either way, likely due to low variability of the nuclear sequence data and thus poor mixing of the reversible-jump Markov chain Monte Carlo (MCMC) algorithms. The small number of sampled loci and individuals for some of the candidate species may also have negatively affected the analysis [58]. Hence, we applied the GMYC model to the mitochondrial data because it does not rely on multiple unlinked loci for delimiting independently evolving lineages. In addition, this method does not require *a priori* assignment of candidate species or specification of guide tree.

The GMYC model optimizes the set of nodes on an ultrametric phylogeny that specify transitions between branching events corresponding to lineage divergence within species and diversification between species [59]. Assignment of genetic clusters is performed under a ML framework by selection of threshold time modeling waiting intervals according to coalescent or Yule processes, respectively. A likelihood ratio test is then conducted to determine if the model of delimited species has a better fit to the data than the null model of a single species. We used the single-threshold version of the ML method implemented in the R package ‘splits’, and the time-calibrated MCC tree estimated in BEAST using unique mtDNA haplotypes was input as the ultrametric phylogeny required by the model.

Because inference of species clusters relies on point estimates of the topology and branch lengths, the associated phylogenetic error could decrease the accuracy of delimitation results. Alternatively, we assessed uncertainty in phylogenetic tree estimation and model parameters with a Bayesian implementation of the GMYC model, which integrates over these potential sources of error via MCMC simulation [60]. We used the R package ‘bGMYC’ to calculate marginal posterior probabilities of species limits from the posterior distribution of ultrametric trees reconstructed with BEAST. A post-burn-in sample of 100 trees resampled from that posterior was used to calculate the posterior distribution of the GMYC model. We adjusted acceptance rates of the MCMC proposals by setting the scale vector to c(1, 20, 0.5). The vector of starting parameters for the model was set to c(1, 0.5, 5) and priors on parameters py2 and t2 were set to 1.2 and 50, respectively. We ran the bGMYC analysis for 100,000 generations, with a burn-in of 90,000 generations and a thinning interval of 100 samples.

### Species tree and divergence time estimation

We jointly estimated the species tree and divergence times for the *T. semitaeniatus* species group based on independent partitions (1 mtDNA and 2 nuDNA loci) under a coalescent model using *BEAST v1.7.5 [48]. We employed a Yule tree prior with uncorrelated lognormal relaxed clocks that allow for rate heterogeneity among lineages and used the same prior scheme for the global substitution rate of the mtDNA described above to calibrate divergence time estimates. For the nuclear loci, substitution rates were estimated relative to the mtDNA rate using a gamma prior for ucld.mean with default values, and exponential prior for ucld.stdev, with a mean of 0.5. Individuals were assigned to species based on the overall structure of the mtDNA gene tree, in which case well-supported haploclades were mostly congruent with the geographic subdivision seen in the SFR valley, resulting in a total of ten lineages for the ingroup (see Results). This is a common approach for the assignment of groups with taxonomic uncertainty [9,61]. In addition we performed *BEAST analyses using a more inclusive species assignment of five evolutionary entities within the *T. semitaeniatus* species group identified by the GMYC. For model selection we compared the fit of different species tree models using Bayes factors calculated from the marginal likelihood estimates [62] with Tracer v 1.6 [49].

We performed five independent runs of 200 million generations each, sampled at every 20,000 steps, totalizing a posterior distribution of 10,000 trees per run. We checked for stationary posterior distributions, ESS above 200, and convergence between runs by examining parameter traces with the program Tracer v1.6 [49]. We combined runs and trees after removing a 10% burn-in with Log Combiner v1.7.5 [48]. We then annotated the combined tree files with TreeAnnotator v1.7.5 [48] to calculate the MCC tree.

### Continuous spatiotemporal phylogeographic reconstruction

The *T. semitaeniatus* species complex is comprised of saxicolous lizards that are currently recognized as either *T. semitaeniatus* or *T. jaguaribanus* (see Results). We employed the mtDNA data set to reconstruct the phylogeographic history of this complex under a continuous spatiotemporal Bayesian approach with BEAST v1.7.5 [48]. Continuous spatiotemporal methods were initially developed for inference of viral epidemics through time [63,64], and more recently have been applied to study diffusion dynamics of slower evolving organisms [30,65,66] and linguistics [67]. Time-homogeneous spatial diffusion models assume a constant Brownian motion between discrete or continuous locations assuming homogeneous diffusion rates across the entire phylogeny [68]. This can be an unrealistic assumption for heterogeneous landscapes settings with major geographic barriers, such as the Caatinga biome and the SFR, respectively. Therefore, we applied a lognormal relaxed random walk (RRW) model, which is a time-heterogeneous approach that allows for variation in diffusion rates across the branches of the phylogeny [63]. By doing so, we were able to infer the geographic location of ancestors and the diffusion of lineages continuously over space and time while accommodating for genealogical uncertainty. We enforced the Jitter option on *multivariateTraitLikelihood* to add a random noise to samples with identical coordinates. We used a skyride model as demographic prior for RRW analyses to estimate effective population sizes through time [69] and the same set of priors for the mtDNA substitution rate as described above. We ran the RRW model including all individuals of the *T. semitaeniatus* complex (i.e., morphologically assigned to *T. jaguaribanus* or *T. semitaeniatus* lizards; n = 123). In addition, we inferred demographic dynamics for each of the three main lineages within the broadly distributed clade of the *T. semitaeniatus* complex (i.e., Clade C; see Results) using Gaussian Markov random field (GMRF) skyride method [69].

We performed five independent runs of 200 million generations each sampled at every 20,000 steps. We assessed parameter traces with Tracer v1.6 [49], combined runs and trees after removing a 10% burn-in with Log Combiner v1.7.5 [48], and annotated tree files and computed the MCC tree with TreeAnnotator v1.7.5 [48]. Subsequently, we generated a visual representation of the spatiotemporal diffusion of lineages in Google Earth using the Continuous Tree module in SPREAD v1.0.7 and Time Slicer to summarize the variation in diffusion rates over time [70].

### Gene flow estimation

We assessed the role of the SFR as a potential barrier to gene flow between geographic populations within the widespread clade of the *T. semitaeniatus* complex (i.e., Clade C; see Results), which are separated into the south or north of the present-day course of this river. Migration parameters were estimated across all loci in terms of mutation-scaled immigration rates *M* (*m*/μ) and mutation-scaled effective population sizes Θ (4*N*_e_μ) were calculated for each population, as implemented in Migrate-n v3.6 [71]. We used a Bayesian approach and thermodynamic integration of four chains with a static heating swap scheme (temperatures: 1.0, 1.5, 3.0, 10^6^), sampling at every 100^th^ increment for a total of 50,000 steps and a burn-in of 50,000 steps.

## Results

### Genetic diversity, gene trees and haplotype networks

We obtained a total of 931 base pairs (bp) for the mtDNA genes and 987 bp for the nuclear markers (Table 3). Nucleotide and haplotype diversity for the combined mtDNA data set was 5.86% and 0.99, respectively. The nuclear markers had an overall low genetic variability, with PDC presenting higher nucleotide and haplotype diversity than BDNF (Table 3). Such low variability reflects the occurrence of shared haplotypes between species of the *T. semitaeniatus* group, in which only *T. pinima* had exclusive haplotypes for the BDNF nuDNA marker, while *T. semitaeniatus* shared some haplotypes with *T. jaguaribanus* and *T. helenae* for both markers (Additional file 1: Figures S1 and S2, Supporting Information).

**Table 3:** **Genetic diversity metrics for the** ***Tropidurus semitaeniatus*** **species group, outgroups, and main mtDNA lineages**

**Gene**	**Length (bp)**	**N/localities**	**H/Hd**	**θ**_***w***_	**Pi (%)/k**	**S**
All individuals						
Cytb	402	134/51	91/0.989	29.691	8.655/34.792	162
16S	529	137/50	67/0.978	22.946	3.746/19.815	123
mtDNA combined	931	138/54	101/0.990	52.637	5.865/54.607	285
BDNF (phased dataset)	564	232/48	59/0.715	13.617	0.331/1.867	82
PDC (phased dataset)	423	230/49	65/0.933	10.477	1.058/4.476	63
mtDNA polymorphism for the main lineages identified						
*T. pinima*						
	931	7/3	6/0.952	15.510	1.888/17.333	38
*T. helenae*						
	931	5/2	3/0.700	11.105	1.432/13.300	23
Seridó						
	931	2/1	1/0.000	0.000	0.000/0.000	0
Serra das Confusões						
	931	3/1	2/0.667	0.667	0.072/0.667	1
Uruçui-Una						
	931	2/1	2/1.000	1.000	0.108/1.000	1
Northern Ceará						
	931	4/2	3/0.833	7.091	0.704/6.500	13
*T. jaguaribanus*						
	931	15/3	6/0.648	7.317	0.583/5.384	23
Northwest						
	931	36/15	30/0.989	19.327	1.591/14.705	79
Northeast						
	931	24/5	8/0.775	52.637	5.865/54.607	285
South						
	931	37/20	30/0.988	24.563	1.928/17.791	102

N = number of samples for a given marker, H = number of haplotypes, Hd = haplotype diversity; θ_*w*_ = Watterson’s theta per sequence, Pi = Nucleotide diversity (per site); k = average number of nucleotide differences between sequences; S = number of polymorphic (segregating) sites.

**Figure 2:**
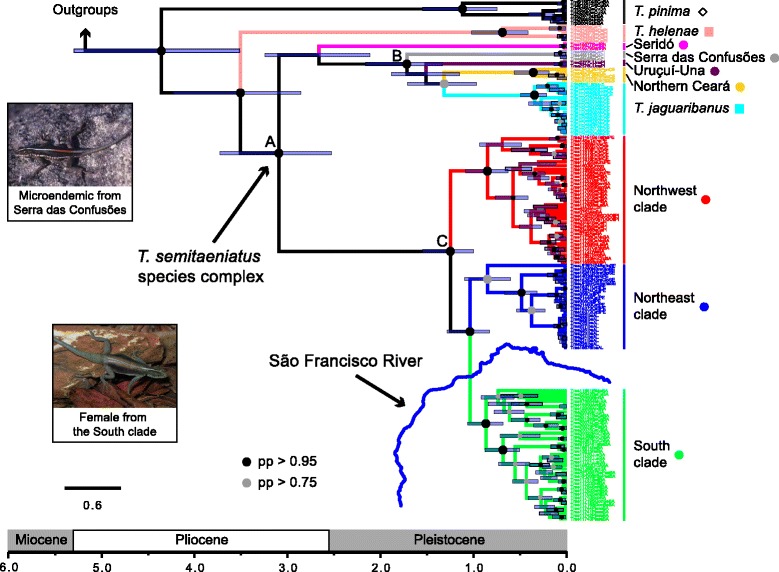
*Tropidurus semitaeniatus* species group mtDNA maximum clade credibility Bayesian tree and divergence dates. Clade colors correspond to localities colors in Figure 1. Posterior probability (pp) values are indicated by node colors and nodes with no indicative of support have pp < 0.75. Photos by MTR.

The mtDNA gene tree is deeply structured with high support for most crown clades and overall congruent between Bayesian (Figure 2) and maximum likelihood (Additional file 1: Figure S1) inferences. *Tropidurus pinima* was recovered as the sister species of all other taxa within the species group, and *T. helenae* formed a sister-group relationship with the clade containing *T. semitaeniatus* and *T. jaguaribanus* species (Clade A). Considering the current taxonomy and the mtDNA gene tree, *T. semitaeniatus* is paraphyletic with respect to *T. jaguaribanus. Tropidurus jaguaribanus* is a recently described species from the Jaguaribe valley that is nested within a clade (Clade B) including divergent lineages attributed to *T. semitaeniatus* on the basis of morphology. In addition to *T. jaguaribanus*, Clade B is comprised of *T. semitaeniatus* from northern Ceará state (localities #1 and #2), Uruçuí-Una (locality #12), and Serra das Confusões (locality #18), whereas *T. semitaeniatus* from Seridó (locality #4) is the sister of Clade B (Figures 1 and 2). All these five lineages have restricted geographic distributions and are designated here as microendemics. The more broadly distributed clade (Clade C) of *T. semitaeniatus* is geographically structured in three different subclades separated by the SFR: the Northwest and Northeast haploclades located on the left riverbank and the South haploclade on the right bank of the river. However, two localities south of the SFR (namely, Nossa Senhora da Glória #34 and Serra de Itabaiana #35, both from Sergipe state) were grouped within the Northeast haploclade (Figures 1 and 2). We here refer to Clade A as the *T. semitaeniatus* species complex.

The Northeast and the Northwest lineages had the higher values of genetic (θ_*w*_) and haplotype (Hd) diversity, respectively (Table 3). Corrected mtDNA distances among microendemic lineages range from 3.8% (between Uruçuí-Una and Northern Ceará) to 7.9% (between Seridó and Serra das Confusões), whereas the distances among those lineages within the broadly distributed Clade C are around 3.5%. Genetic distances between any lineages from each of the two major clades within the *T. semitaeniatus* species complex are all higher than 8.6% (Table 4).

**Table 4:** **Average among groups’ genetic distances between major sampled clades for the mtDNA data**

	**Ediv**	**This**	**Ttor**	**Tpin**	**Thel**	**TsSer**	**TsS**	**TsNE**	**TsNW**	**TsSCo**	**TsNoCE**	**Tjag**	**TsUUn**
Ediv		0.142 (0.015)	0.145 (0.015)	0.142 (0.014)	0.147 (0.014)	0.157 (0.015)	0.148 (0.014)	0.158 (0.014)	0.155 (0.014)	0.155 (0.014)	0.163 (0.015)	0.163 (0.015)	0.158 (0.014)
This	0.127 (0.012)		0.021 (0.005)	0.118 (0.012)	0.121 (0.013)	0.136 (0.015)	0.129 (0.014)	0.135 (0.014)	0.116 (0.013)	0.140 (0.015)	0.141 (0.015)	0.140 (0.015)	0.133 (0.014)
Ttor	0.130 (0.012)	0.021 (0.005)		0.111 (0.012)	0.123 (0.013)	0.140 (0.015)	0.125 (0.013)	0.130 (0.014)	0.116 (0.013)	0.140 (0.014)	0.136 (0.014)	0.131 (0.014)	0.127 (0.013)
Tpin	0.128 (0.011)	0.106 (0.010)	0.101 (0.010)		0.105 (0.011)	0.114 (0.012)	0.118 (0.011)	0.122 (0.013)	0.119 (0.012)	0.108 (0.011)	0.110 (0.011)	0.110 (0.011)	0.106 (0.011)
Thel	0.132 (0.011)	0.110 (0.011)	0.112 (0.010)	0.096 (0.009)		0.092 (0.011)	0.095 (0.010)	0.105 (0.012)	0.096 (0.010)	0.097 (0.011)	0.096 (0.011)	0.094 (0.011)	0.088 (0.010)
TsSer	0.140 (0.012)	0.122 (0.012)	0.124 (0.011)	0.104 (0.010)	0.084 (0.009)		0.089 (0.010)	0.088 (0.011)	0.091 (0.010)	0.079 (0.010)	0.077 (0.009)	0.080 (0.010)	0.076 (0.010)
TsS	0.133 (0.011)	0.115 (0.011)	0.112 (0.011)	0.106 (0.009)	0.087 (0.008)	0.081 (0.008)		0.030 (0.004)	0.036 (0.005)	0.088 (0.010)	0.088 (0.010)	0.091 (0.010)	0.087 (0.010)
TsNE	0.140 (0.011)	0.120 (0.011)	0.115 (0.011)	0.109 (0.009)	0.094 (0.009)	0.081 (0.009)	0.029 (0.004)		0.036 (0.005)	0.091 (0.011)	0.085 (0.010)	0.088 (0.011)	0.086 (0.011)
TsNW	0.138 (0.011)	0.105 (0.010)	0.105 (0.010)	0.107 (0.009)	0.088 (0.008)	0.084 (0.009)	0.035 (0.005)	0.035 (0.005)		0.096 (0.011)	0.088 (0.010)	0.091 (0.010)	0.086 (0.010)
TsSCo	0.139 (0.011)	0.124 (0.011)	0.124 (0.011)	0.099 (0.009)	0.089 (0.009)	0.074 (0.008)	0.081 (0.009)	0.083 (0.009)	0.087 (0.009)		0.054 (0.008)	0.059 (0.009)	0.045 (0.007)
TsNoCE	0.145 (0.012)	0.126 (0.011)	0.121 (0.011)	0.101 (0.009)	0.087 (0.009)	0.072 (0.008)	0.081 (0.008)	0.078 (0.009)	0.081 (0.008)	0.051 (0.007)		0.044 (0.007)	0.038 (0.006)
Tjag	0.145 (0.012)	0.125 (0.011)	0.117 (0.011)	0.100 (0.009)	0.087 (0.009)	0.075 (0.009)	0.083 (0.009)	0.081 (0.009)	0.083 (0.009)	0.056 (0.008)	0.043 (0.006)		0.046 (0.007)
TsUUn	0.141 (0.011)	0.119 (0.011)	0.114 (0.010)	0.097 (0.009)	0.081 (0.009)	0.071 (0.008)	0.080 (0.008)	0.080 (0.009)	0.079 (0.008)	0.043 (0.007)	0.037 (0.006)	0.044 (0.007)	

Values below the diagonal are uncorrected p-distances and values above the diagonal are corrected p-distances using the Tamura-Nei model [56], followed by the respective standard errors, calculated using 500 bootstrap replicates. Analyses were conducted in in MEGA5 [55]. Ediv = *Eurolophosaurus divaricatus* (OG); This = *T. hispidus* (OG); Ttor = *T. torquatus* (OG); Tpin = *T. pinima;* Thel = T. *helenae*; TsSer = *T. semitaeniatus* from Seridó, Rio Grande do Norte state; TsS = *T. semitaeniatus* South of the SFR; TsNE = *T. semitaeniatus* Northeast of the SFR; TsNW = *T. semitaeniatus* Northwest of the SFR; TsSCo = *T. semitaeniatus* from Serra das Confusões, Piauí state; TsNoCE = *T. semitaeniatus* from North of Ceará state; Tjag = *T. jaguaribanus*; TsUUn = *T. semitaeniatus* from Uruçui-Una, Piauí state.

Although the PDC gene tree has low resolution and nodal support, there can be noted yet some geographic structuring in the tree and haplotype network, whereas the BDNF gene tree lacks resolution and support for the great majority of nodes (Additional file 1: Figures S1 and S2). There is substantial haplotype sharing across geographic regions for the two nuclear loci, indicating insufficient time for complete lineage sorting since population divergence. The most frequent (52.2% of individuals) and widely distributed haplotype in the BDNF network is shared among *T. semitaeniatus*, *T. jaguaribanus* and *T. helenae* (Additional file 1: Figure S2). Additionally, some of the microendemic lineages have exclusive haplotypes; for example, Uruçuí-Una for PDC and Serra das Confusões for both PDC and BDNF (Additional file 1: Figure S2).

### Species delimitation

**Figure 3:**
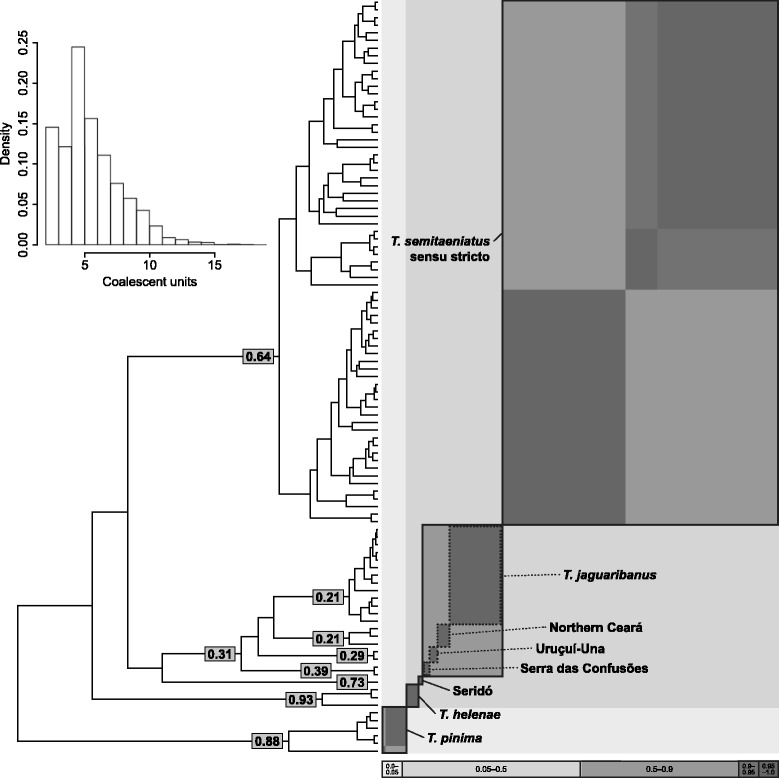
Species delimitation based on the Generalized Mixed Yule Coalescent model. Summary of species delimitation analyses using maximum likelihood and Bayesian implementations of the Generalized Mixed Yule Coalescent model for saxicolous lizards of the *Tropidurus semitaeniatus* species group. The phylogeny is the maximum clade credibility tree from BEAST. The five ML entities identified by the GMYC method are outlined with continuous contours and microendemic lineages (expect for Seridó) are depicted with dashed lines. Numbers are the posterior probability of species identities calculated from a posterior distribution of trees generated in BEAST. The histogram represents uncertainty in coalescent units recovered in bGMYC analysis and grayscale plot is a sequence-by-sequence matrix colored by pair-wise posterior probabilities of conspecificity, where off-diagonal patterns indicate uncertainty of species limits owing to topological variation of phylogenetic tree.

The GMYC maximum likelihood analysis recovered five evolutionary entities (Figure 3) with a confidence interval of 1–17 genetic clusters and a non-significant model of species delimitation (*P* = 0.41). Nodal support for delimited species, defined as the sum of Akaike weights of candidate delimitation models in which the node is included, was less than 0.5 within a 95% confidence set of 47 out of 98 models compared. The mean number of evolutionary entities delimited by the bGMYC analysis was 5.91 (mode = 5), with a 95% HPD probability interval of 2–10 species clusters. Four of the five maximum likelihood entities match those coalescent units with the highest marginal probabilities, whereas microendemics from Serra das Confusões showed higher marginal probability than the genetic cluster including all microendemic lineages but Seridó. However, none of the most frequently recovered species limits was found in greater than or equal to 95% of the posterior distribution (Figure 3).

### Species tree and divergence times

**Figure 4:**
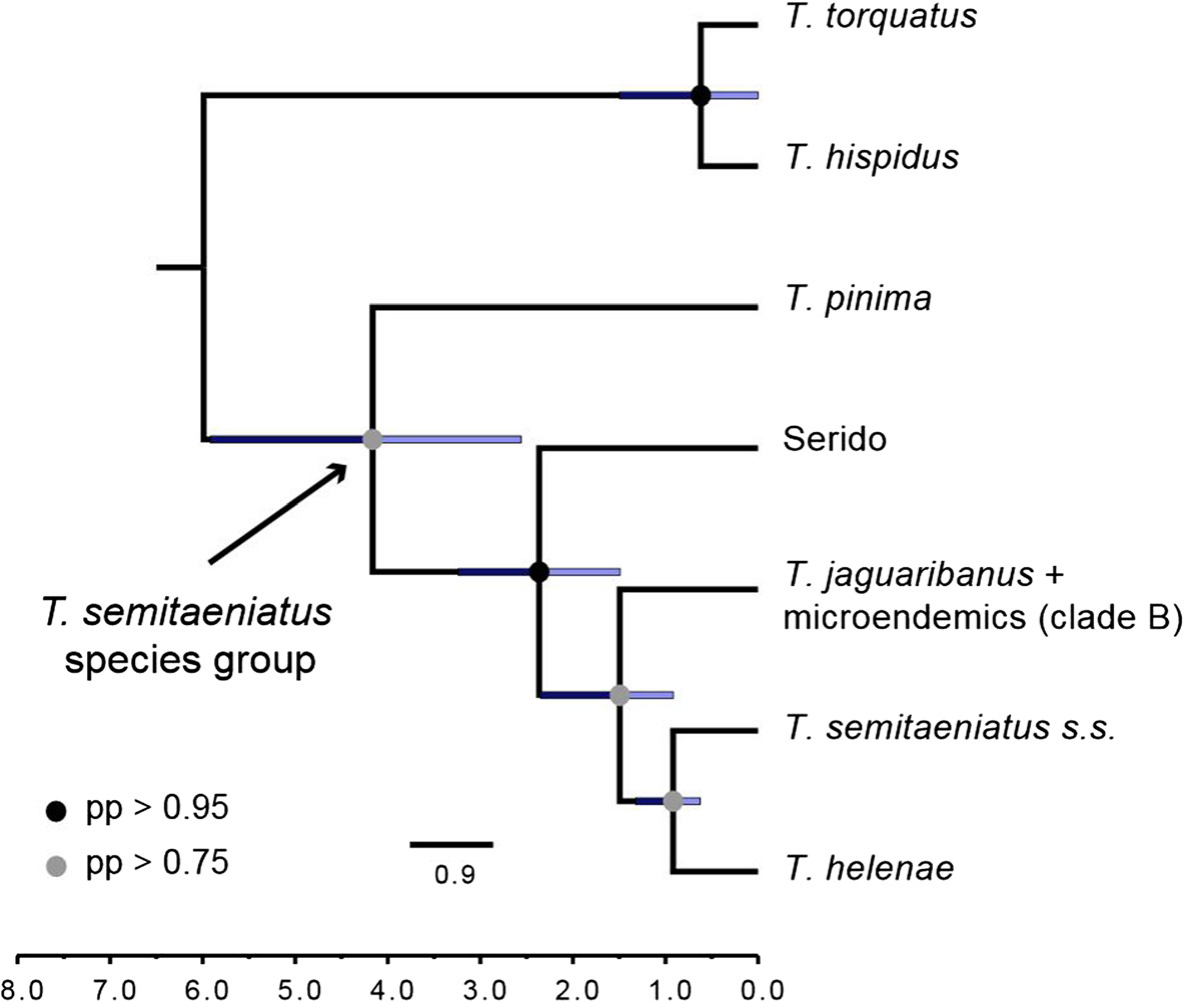
Species tree and divergence times for the *Tropidurus semitaeniatus* species group and outgroups based on the more inclusive species assignments from GMYC. The maximum clade credibility tree was inferred under a coalescent model based on all three independent loci with *BEAST. Posterior probability (pp) values are indicated by colored circles in the node colors; nodes with no indicative of support have pp < 0.75.

The species tree estimation using the assignment of mtDNA haploclades recovered interspecific relationships that are similar to those on the mtDNA gene tree, except for the placement of Seridó microendemics as sister to the three broadly distributed lineages comprising Clade C, albeit with low nodal support (Additional file 1: Figure S3). Alternatively, the species tree based on the assignment of genetic lineages detected by the GMYC species delimitation had higher support for all nodes and was favored by a Bayes factor (logBayes = 37.79) indicating strong support for the latter hypothesis. In this species tree, *Tropidurus pinima* is sister to a clade containing all other taxa in the species group, including *T. helenae* nested within the *T. semitaeniatus* species complex (Figure 4). However, the position of *T. helenae* was weakly supported. This suggests that although the species tree may recover the main relationships, some degree of topological uncertainty still remains, specially with respect to *T. helenae*, and additional markers would be required to solve this matter.

Divergence dates on the species tree (Figure 4) are slightly younger than time estimates on the mtDNA gene tree (Figure 2). The initial diversification of the species group, as inferred from the mtDNA data set, took place at 4.38 million years ago (Ma) with a 95% highest posterior density (HPD) interval ranging from 5.3 to 3.5 Ma. The *T. semitaeniatus* species complex diverged at 3.1 Ma (95% HPD: 3.7–2.5 Ma), while the microendemic lineages from Seridó or nested within Clade B and the broadly distributed Clade C diverged at 2.6 Ma (95% HPD: 3.2–2.1 Ma), 1.7 Ma (95% HPD: 2.2–1.3 Ma) and 1.2 Ma (95% HPD: 1.5–1.0 Ma), respectively (Figure 2). On the other hand, according to the species tree (Figure 4), diversification of the species group began at 4.2 Ma (95% HPD: 5.9–2.6 Ma), whereas the species complex (including *T. helenae*) diverged at 2.4 Ma (95% HPD: 3.2–1.5 Ma), and Clade B at 1.55 Ma (95% HPD: 2.35–0.92).

### Spatiotemporal diffusion, population size changes, and migration rates

**Figure 5:**
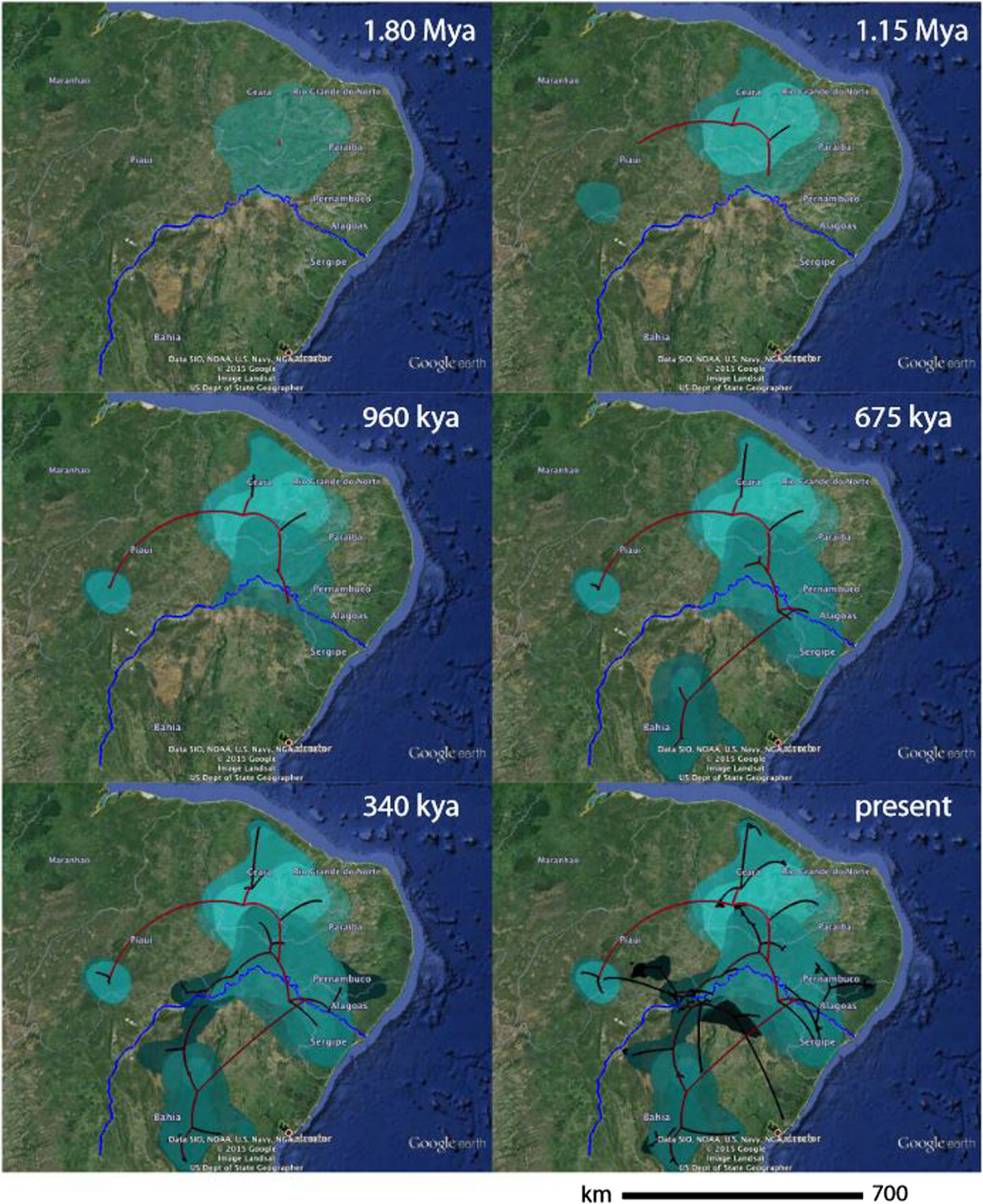
Bayesian spatiotemporal diffusion of *Tropidurus semitaeniatus* complex at 6 time slices. Reconstructions are based on the maximum clade credibility tree estimated with a time-heterogeneous Relaxed Random Walk (RRW) Bayesian phylogeography approach. Shading represents 80%-HPD uncertainty in the location of ancestral branches with lighter and darker shades representing older and younger diffusion events, respectively.

The RRW diffusion model inferred the geographic origin of the *T. semitaeniatus* species complex at approximately 180 km north of the current course of the SFR, in the limits between Ceará and Paraíba states (latitude: -7.18, longitude: -38.76; Figure 5). The spatiotemporal reconstruction indicates that the *T. semitaeniatus* species complex diverged at 1.94 Ma (95% HPD: 2.42–1.50) and experienced four main colonization phases: (1) an initial northwestward dispersal with establishment of the ancestors of the northern Ceará and *T. jaguaribanus* microendemic lineages by 1.15 Ma; (2) two long-distance dispersals towards the south at around 960 ka, with the Uruçui-Una and Serra das Confusões microendemics in one front of colonization into the Parnaíba valley region and the more broadly distributed *T. semitaeniatus* lineage within close proximity to the SFR in the other front, when the first lineage appeared very close to the current right bank of the river; (3) after traversing the river, *T. semitaeniatus* split into a lineage alongside the lower course of the present-day SFR and another that underwent a long-distance dispersal towards southern Bahia state; (4) within the last 600 ka, microendemics reached their current distributions and the Northwest *T. semitaeniatus* lineage dispersed further south bounded by the left border of the SFR, while the southern counterpart spread northwardly until reaching the right margin, expect in Sergipe state where it crossed to the left riverbank (but see Discussion).

**Figure 6:**
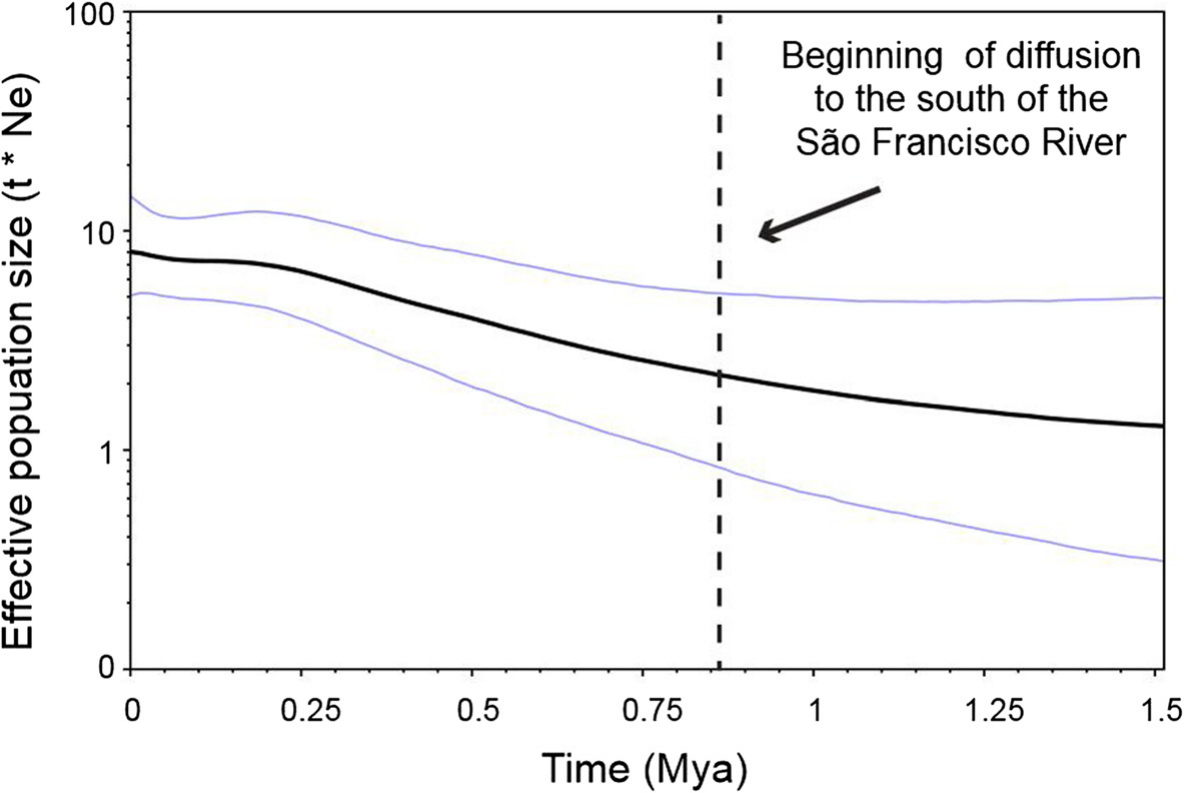
T. *semitaeniatus* complex effective population size through time based on the Bayesian Skyride. Area delimited by the blue line represented the 95%-HPD interval.

The mean diffusion rate for the *T. semitaeniatus* complex across the RRW reconstruction was 294.5 km per million years (95% HPD: 244.4–347.6 km/Myr), but varied across time slices. For instance, dispersal rates were higher in the last million years, with a mean of 483 km/Myr. This increase in dispersal rates coincides with an increase in population size, as detected in the Bayesian Skyride analysis (Figure 6), and the long-distance dispersals to the south of the São Francisco drainage system, which is indicative of simultaneous demographic growth and range expansion. When the three main lineages within Clade C were analyzed separately, we detected population size increases both in the South and Northwest, but no signal of historical fluctuations were observed in the Northeast (Additional file 1: Figure S4).

We detected non-zero migration rates across the SFR in both directions; however, the number of immigrants per generation into the northern population (4 *Nm*_north_ = 7.2397) was almost three times higher than into the southern population (4 *Nm*_south_ = 2.4556). Additionally, the mutation-scaled effective size of the northern population (Θ _north_ = 0.1307) was more than five times larger than that of the southern population (Θ _south_ = 0.0252). Effective sample sizes were above 1,000 for all estimated parameters Θ and *M*, including the likelihood of genealogies, as recommended by the program manual.

## Discussion

Saxicolous lizards of the *Tropidurus semitaeniatus* species group are endemic and adapted to the Brazilian Caatinga, representing some of the most conspicuous faunistic elements in the biome. Here we generated sequence data for three independent markers from *T. semitaeniatus* species group lizards spanning most of the Caatinga and employed a molecular phylogenetic approach to investigate species relationships, their diversification history, and the role of the São Francisco River as a biogeographic barrier for this group. We found evidence for the occurrence of cryptic evolutionary lineages with high mtDNA genetic distances and strong geographical structure with respect to this major perennial river in the semiarid Caatinga. However, despite such high genetic diversity we did not find strong support for accurate species delimitation.

### Phylogenetics and historical biogeography

Phylogenetic reconstructions inferred for the *T. semitaeniatus* species group using gene trees, haplotype networks and species tree analyses recovered similar topological relationships among its species. Analyses recovered with high support *Tropidurus pinima* as the sister to all other species in the group and a *T. jaguaribanus* + *T. semitaeniatus* clade that rendered *T. semitaeniatus* paraphyletic (i.e., the *T. semitaeniatus* complex), unless a broader definition of *T. jaguaribanus* is used. However, the ambiguous placement of *T. helenae* and the Seridó lineage and overall low nodal support of the species tree as compared to the mitochondrial gene tree demonstrate instances of genealogical discordance and phylogenetic uncertainty.

Incomplete lineage sorting and gene flow among populations or species are evolutionary processes notably known to cause gene tree discordance and hamper species tree estimation, especially among populations or closely related species with low levels of divergence [72,73]. The relatively short intervals between speciation events on the species tree, together with the low variability and haplotype sharing of nuclear markers, suggest that such discordance may likely represent some retention of ancestral polymorphisms due to incomplete lineage sorting. However, the possibility that gene flow has an effect on the phylogenetic inference cannot be discarded since we found evidence of migration between populations separated by the SFR, and thus introgression might also be the case involving other taxa in the species group. Nevertheless, despite the overall low support of the species tree, our data had sufficient signal to reconstruct a time-calibrated phylogeny for the *T. semitaeniatus* species group with acceptable divergence date estimates and credibility intervals. While we advance the most complete phylogenetic hypothesis of interspecific relationships within this group to date, increasing the number of individuals sampled per lineage across its range in combination with additional informative markers is necessary to assess the impact of ancestral polymorphism and gene flow on species tree resolution.

The initial divergence of the *T. semitaeniatus* species group took place in the Late Miocene–Pliocene transition. Geomorphological studies suggest that the SFR had different paleocourses before reaching its present-day configuration. Although the paleo-SFR characterized by an outflow into the equatorial Atlantic is a feature too ancient [31] to have prompted diversification within this group, the paleogeographic setting in place during the post-rift history of NE Brazil until the Late Miocene seems to have set the stage for the early differentiation of *T. semitaeniatus* stem lineages. Multiple episodes of local uplift with subsequent erosion and deposition have shaped the landscape of the Brazilian northeast during development of the continental margin and adjacent hinterland [74,75]. These events were coeval with a period of lateritic soil formation after the last Miocene marine transgression [76], a regional crustal cooling consistent with intensified topographic erosion [77], and a global climate trend towards lower temperatures [78]. In addition, episodic changes associated with intraplate tectonics happening well into the Quaternary [76] likely played an important role in the evolutionary history of the *T. semitaeniatus* species group, within which most crown clades diverged more recently in the Pleistocene.

The onset of the Northern Hemisphere glaciation beginning in the Late Miocene and its intensification with build-up of ice sheets by the Late Pliocene [79] must have contributed significantly to the expansion of a semiarid climate in NE Brazil. Accordingly, in addition to locally reactivated topography due to neotectonics [80], development of a regional lower erosional surface at the expense of elevated plateaus [74] must have driven divergence of *T. semitaeniatus* species as well as population structuring during Plio-Pleistocene times. The presence of thick paleodunes fields in the area of the middle SFR also indicates prevalence of semiarid conditions across the Caatinga region possibly dating as far back as the late Tertiary [36]. Early geomorphological studies suggested that dune-prone sites would have climaxed during the last glacial stage at ~18,000 B.P. [33,34]. Based on this view Rodrigues [27] proposed the paleolacustrine hypothesis, according to which at the time of the Würm-Wisconsin glaciation the SFR acquired an endorheic drainage pattern that would have promoted allopatric divergence of saxicolous lizards from disjunct inselbergs and high surfaces. On the other hand, the drainage might have reached its current exorheic pattern and found its way into the southeastern Atlantic Ocean already during the Middle Pleistocene at the end of the Mindel glaciation [32].

Alternatively, Rodrigues [27,35] admitted the possibility that the SFR could have occupied forsaken meanders that would likewise act as an isolating barrier to the herpetofauna. While Quaternary glaciations exacerbated the typical semiarid climate of the Caatinga biome and likely the phylogeographic structure of *T. semitaeniatus* lizards, such paleoclimatic events are rather recent to account for lineage diversification at deeper levels entirely. Perhaps, a paleocourse to the south of the present mouth (Figure 1c) may explain the divergence between populations of *T. semitaeniatus* separated by the mid-lower section of the SFR. Interestingly, downstream of Paulo Afonso Dam there are several transcurrent and transpressional fault zones located in the southern part of the Borborema Province, between Sergipe and Alagoas states [81], that might be implicated in the control of paleochannels formerly connected to the Atlantic possibly through the Vaza-Barris area in the Early Pleistocene. Two localities from Sergipe (#34 and 35) located south of the current SFR, but north of the supposedly forsaken meanders, are grouped with the Northeast clade of the mtDNA gene tree. This instance of genealogical discordance may be linked to a final shift on the mid-lower course of the SFR, indicating that the river did not act as a vicariant barrier between these areas in the recent past. Historical gene flow and incomplete lineage sorting are then likely processes to have shaped this observed pattern. Our spatially explicit phylogeographic inference and migration rate estimates give further evidence for the past and current roles of the SFR as a driver of genetic diversity and population structure within this group.

The deep phylogenetic structure, old divergence times (~2.4 Ma on the species tree and 3.1 Ma on the mtDNA tree), and large genetic distances are indicative that *T. semitaeniatus*, previously considered widespread, is actually a species complex comprised of several lineages, some of which are microendemics. Microendemic lineages from Seridó, Northern Ceará, Uruçuí-Una, and Serra das Confusões are as yet unrecognized taxonomically, but do represent unique evolutionary units that deserve taxonomic reassessment (see below). The long branches leading to these microendemic taxa might have resulted from extinction of sister lineages [82], a plausible scenario if rocky habitats were eroded and became scarce in the intervening regions [20], given the site specificity of *T. semitaeniatus* group to saxicolous environments.

The distribution of microendemic lineages identified here add evidence to the body of knowledge that indicates the encompassing regions as important regional centers of diversification within the Caatinga biome that should be prioritized for conservation [83]. For example, the Seridó region, which mostly overlays with the Sertaneja Depression and the Borborema Plateaus domains (Figure 1) and have strong influence of Precambrian crystalline basements and substantial occurrence of inselbergs, have been identified as a noteworthy regional refugia for a rich and endemic biota [84]. Likewise, occurrence of the endemic lineages from Northern Ceará at the margin of the Central Ceará Highlands and Jaguaribe river valley endorses the complex geomorphology reported for this region [20], harboring more than one endemic lineage in the group, as the closely related *T. jaguaribanus* is also restricted to this region. The Jaguaribe river valley area is delimited by the Ibiapaba, Araripe and Borborema plateaus that were shaped by Miocene uplifts [20,77]. Other endemic species [41,85] and species level relationships [86] also support vicariant events isolating the Jaguaribe river valley and adjacent drainages. Finally, lineages from Uruçuí-Una and Serra das Confusões, both located in Piauí state, despite being geographically close (~94 km) are separated since ca. 1.7 Ma by long branches and show prominent mtDNA distances (4.3%) that indicate independent evolutionary histories. These two regions are also remarkable for their geographic location in a complex and narrow contact zone between the Caatinga and Cerrado biomes, where intense erosional processes presumably have isolated patches of rocky habitats that reduced gene flow and promoted allopatric divergence. For example, the much older Serra das Confusões lineage is in close proximity to a recent newcomer of the northwest clade from Morrinhos (locality #14). Serra das Confusões National Park is located on top of a plateau drained by channels flowing northwards to the Parnaíba river and has some unique geomorphological formations characterized by highly dissected mountain ranges with round tops and associated interplateau valleys [87,88].

The importance of Neotropical inselbergs and elevated plateaus in offering ‘sky-islands’ for diversification of endemic biota has been explored in bromeliads adapted to such habitats in the Atlantic Forest of southeastern South America [25,89,90]. Here we provide evidence that unique lineages within the *Tropidurus semitaeniatus* species group are the result of allopatric speciation events in disjunct highlands, which are typically older than lower-surface pediplains in NE Brazil. Presence of multiple endemic lineages of *T. semitaeniatus* seem to agree with the occurrence of climatic dynamism in the Caatinga, with both stable and unstable areas of SDTFs [91]. Stability of isolated rocky surfaces must have favored the diversification of this species group, but was probably not enough to cease gene flow completely as long as surrounding areas were covered by open-dry formations with compatible thermal preferences. Thus, a scenario of partial isolation produced by erosion and accompanied by forest isolation during humid periods seems plausible. In addition, in agreement with results reported for other species-rich regions such as the Atlantic Coastal Forest [66,92] and the Australian Wet Tropics, population divergence occurred at small geographic scales in the Caatinga where stable rocky areas would have acted as local refugia [93] within the semiarid Caatinga nucleus at the continental scale [91].

In summary, two large drainage basins correspond to the main patterns of geographic structuring in the *T. semitaeniatus* complex: microendemic lineages located at the northern range (Seridó, *T. jaguaribanus*, and Northern Ceará) are distributed in the Jaguaribe and Piranhas-Açu river basins that flow into the northern coast of Brazil, while the three wide-ranging lineages (Northwest, Northeast, and South clades) are associated with the São Francisco river basin [20]. Further structure is found among lineages associated to the São Francisco drainage (see below).

The large mitochondrial genetic distances among major lineages are comparable to among-species distances reported for other tropidurid lizards [29,94] and are suggestive of cryptic genetic diversity within the *T. semitaeniatus* species group. Given the long-term semiarid climate regime of NE Brazil allied to its intricate geological history, it is possible that these lineages indeed represent independently evolving taxa. Nevertheless, the application of a universal cutoff is problematic because of variation in substitution rates and effective population sizes among lineages, such that distance-based metrics should be taken with caution and interpreted in combination with other methods for species delimitation [95]. By applying the GMYC model, we found evidence of five distinct evolutionary entities within the *T. semitaeniatus* species group. Two are valid taxonomic species (*T. pinima* and *T. helenae*), one comprises the more broadly distributed Northwest, Northeast and South lineages (i.e., *T. semitaeniatus sensu stricto*, which would keep the specific status according to the type locality, Sincorá Velho, BA; Figure 1), one represents the microendemic lineage from Seridó, and another includes the microendemics from Serra das Confusões, Uruçuí-Una, Northern Ceará as well as the recently described species from Jaguaribe valley, *T. jaguaribanus* (Clade B).

In that sense, a more inclusive definition of *T. jaguaribanus* in order to include these microendemic lineages could render reciprocal monophyly of three lineages within the *T. semitaeniatus* complex. However, we have morphological evidence based on color patterns reinforcing the distinctiveness of microendemic lineages nested with *T. jaguaribanus* (MTR, *Pers. Obs*.) so we advocate caution with this interpretation. The GMYC analysis had a broad confidence interval and delimited species had low nodal support but still, we were able to detect genetic clusters of all microendemic lineages and possibly within *T. semitaeniatus sensu stricto* divided between north and south by the SFR. A small number of samples per lineage (i.e., less than three), particularly of microendemics, may explain the lack of accurate delimitation of the *T. semitaeniatus* species group using single-locus GMYC [59], although studies indicate that random sampling has a minor effect in the model performance [96] Alternatively, since the *T. semitaeniatus* species group is geographically structured in genetic clusters, we may interpret this uncertainty in threshold times as reflecting relatively high genetic diversity within distinct lineages, large effective population sizes and rapid diversification, conditions that are very challenging for the method in general [60,96]. Because similar limitations were observed in South American geckos associated with rock outcrops of open-dry formations [5], it is likely that under such biogeographic scenarios the GMYC model will not accurately identify cryptic genetic lineages. As a single-locus method, GMYC will only identify mtDNA lineages, which may not agree with species-tree lineages, especially in the light of possible introgression. Therefore, we recommend that the different independently evolving lineages identified here need to be assessed with further coalescent-based studies using more loci and a better taxon sampling across the range of *T. semitaeniatus* lizards. In addition, these independent lineages will require closer examination of ecomorphological data to provide diagnostic characters and taxonomic descriptions that can recognize them as valid species [97].

Systematic revisions of the *Tropidurus semitaeniatus* species group will also require conservation reassessment of its constituent taxa. Wide-ranging species are usually categorized as Least Concern, as is the case for *T. semitaeniatus*, which is listed as single species in the IUCN Red List [98]. Reevaluations of the IUCN threat status have been suggested for the endemic frog *Ischnocnema guentheri*, known only from its the type locality but previously considered widespread [66]. The Caatinga biome is exclusively Brazilian and harbors a distinct biota adapted to semiarid environments [99], with special emphasis to seven endemic species of *Tropidurus* [100]. According to our results, it is possible that the endemism of Caatinga lizards is even higher than previously thought. However, despite strong anthropic and desertification threats, the Caatinga has the smallest percentage of areas under legal protection among any major Brazilian biome [11,101]. Species in the *T. semitaeniatus* group tend to be abundant where they occur [41] and we were able to sample several populations from legally protected areas. As a consequence, species in this group do not seem to be under major population viability threats at present time. Nonetheless, a comprehensive mapping of the distribution limits of the lineages detected here is necessary to identify potential conservation gaps and fully evaluate the threat status of the *T. semitaeniatus* species group.

### Phylogeography of the *T. semitaeniatus* complex and the São Francisco River

The spatiotemporal reconstruction showed that after an origin to the north of the current course of the SFR, the *T. semitaeniatus* complex spread southwards and microendemic lineages were established on the extreme northern range. Diffusion phases indicate a demographic history that started at the beginning of the Pleistocene and was marked by long-distance diffusion events that can be associated with the geomorphology of the Caatinga and the SFR.

It is interesting that the *T. semitaeniatus* complex preserved high genetic diversity during its range expansion. Geographic expansion by short-distance dispersals (SDD) events tend to result in reduced genetic diversity at the expanding front due to sequential founding events and genetic drift, potentially limiting the evolutionary potential at the range margin [102]. Conversely, long-distance dispersals (LDD) events can overcome sequential founding effects and mitigate reductions of genetic diversity associated with range expansion by SDD [102]. LDD can potentially promote adaptive evolution in novel environments and improve the fitness at the range periphery [103]. LDD events during the diversification of *T. semitaeniatus* likely contributed to the maintenance of high mtDNA genetic diversity and retention of ancestral polymorphism in the group.

Although diffusion rates varied across time slices, those estimates were always less than one meter per year. At first glance, this rate seems too low when compared to values published for other lizards. For example, Camargo et al. [104] reported a mean diffusion rate of 1.1 m/year for the lizard *Liolaemus darwinii* and a maximum value of 17 m/year at the beginning of rapid range expansion. However, *T. semitaeniatus* group have low vagility due to their dependency on rocky outcrops, limiting possible dispersal events. Field estimates of home range for *T. semitaeniatus* vary between 1–20 m^2^ and indicate a high philopatry and low dispersal ability for the species (D. Passos, *Pers. Comm*.). Although many other factors influence diffusion patterns over space and time (e.g., different dynamics for invasive and core populations due to evolutionary and ecological innovations at the expanding range [105]; differences between historical and ongoing range shifts [106]), these field observations suggest a good fit of the diffusion rates estimated for the *T. semitaeniatus* complex.

The riverine hypothesis has been traditionally invoked to suggest that major Amazonian rivers play primary roles as drivers of terrestrial vertebrate diversification [107-109]. In addition, non-Amazonian rivers also constitute important historical barriers involved in the biotic diversification of other Neotropical biomes [30,110]. More specifically for the SFR, recent studies explored whether this river influenced patterns of species diversity and population structuring. Nascimento et al. [30] found that speciation of the rodent genus *Thrichomys* occurred during the Late Miocene, when the SFR supposedly followed a different course congruent with the first and more dramatic change of paleocourse (Figure 1a). Moreover, the geographic distribution and phylogenetic relationships of *Thrichomys* suggest the existence of frequent past-connections between both banks in the middle section of the SFR [30]. Likewise, the rodent *Calomys expulsus* [111] and the mousse opossum *Gracilinanus agilis* [112] are geographically structured into lineages from the left and right margins of the SFR, which was pointed as a vicariant barrier to gene flow among populations of these taxa. Evolutionary patterns of the gecko *Phyllopezus pollicaris*, a species complex endemic to the dry diagonal, similarly suggest that the SFR act as a barrier promoting diversification at the intraspecific level [9]. This result also is in accordance with previously described patterns for several lizards at deeper taxonomic levels (i.e., interspecific and intergeneric), in which species-pairs are restricted to sandy habitats at opposite banks, such as the psamophilous genera *Eurolophosaurus*, *Calyptommatus*, and *Nothobachia* [27,29,113]. Our results indicate that the current course of the SFR as well as its paleocourses promoted diversification of the endemic *T. semitaeniatus* species group. Although it could be tempting to assert that frequent dispersion events between both banks were detected, this was probably not the case, given that diffusion events across the SFR may actually have taken place when the river occupied a different course.

The RRW approach is able to distinguish between demographic and range expansion processes, an important feature given that range expansion can occur without population growth, and vice versa [65]. Range expansion and population growth of the *T. semitaeniatus* complex occurred simultaneously, specially when lineages south of the SFR became isolated after its current course was reached in the Late Pleistocene (Figures 1d, 5, 6, and Additional file 1: Figure S4). During this period, southern Bahia lineages (South clade) went through range expansion towards the north, while lineages from the Northwest clade that were isolated to the north of the SFR also expanded their ranges along the left border of the river. As the final outcome, neighbor populations currently located on adjacent but opposite sides of the middle section of the SFR (some as close as 10 km) do not form sister lineages. Instead, these lineages developed independent evolutionary trajectories and probably reached their current distributions after the final course of the SFR was already in place.

If the SFR served as a strong vicariant agent for the ancestor of *T. semitaeniatus sensu stricto* we would expect zero or only negligible gene flow between populations from separate margins. However, we found non-zero migration rates in both directions. Given our spatiotemporal reconstruction and the paleogeographic setting of NE Brazil, we hypothesize that the ancestral population of *T. semitaeniatus sensu stricto* became established in the mid-section of the SFR at around one million years ago, and thence participated in the latest Pleistocene reconfiguration of its paleocourse. In addition, landscape development in the region is mainly controlled by climate and neotectonics, without major fluvial channel alterations in the recent past (i.e., late Quaternary). Therefore, the observed differences in migration rates almost three times more into the northern population may reflect the postulated northward capture of the lower SFR and might be explained by the historical passive transferring of alleles along this stretch.

## Conclusions

Speciation in the *T. semitaeniatus* species group took place during the Pliocene–Pleistocene transition and is intrinsically related to long-term stability of isolated rock surfaces associated with semiarid climatic conditions and differential topographic rearrangements resulting from erosional and depositional processes in addition to tectonics shaping the Caatinga landscape. The evolutionary history of this saxicolous group was marked by diffusion events that support high cryptic genetic diversity and occurrence of microendemic lineages. Two main landscape events that can be implicated in the phylogeographic structure of the *T. semitaeniatus* group in the Caatinga include the establishment of the Jaguaribe and Piranhas-Açu river basins and the final establishment of the São Francisco drainage.

## Availability of supporting data

The data sets supporting the results of this article are included within the article and its supplementary files.

## Additional file

Additional file 1: Figure S1. Mitochondrial DNA (cytb + 16S), BDNF and PDC gene trees for the *Tropidurus semitaeniatus* species group, as estimated by maximum likelihood (ML). Colors correspond to localities and clades colors in Figure 1 and Figure 2. **Figure S2**: *Tropidurus semitaeniatus* species group BDNF and PDC haplotype networks build with HaploViewer. Clade colors are the same as in Figure 1 and 2. Numbers inside the proportionally sized circles represent the number of alleles sharing that particular haplotype. **Figure S3**: Species tree and divergence times for the *Tropidurus semitaeniatus* species group and outgroups based on the less inclusive species assignments from the mtDNA haploclades. The maximum clade credibility tree was inferred under a coalescent model based on all three independent loci with *BEAST. **Figure S4**: Effective population sizes through time based on the Bayesian Skyride as estimated for each of the three main population strains (Clade C) for the T. *semitaeniatus* complex. Area delimited by the blue line represented the 95%-HPD interval.

## Abbreviations

SDTFs: Seasonally Dry Tropical Forests
SFR: São Francisco River
mtDNA: Mitochondrial DNA
nuDNA: Nuclear DNA
BDNF: Brain-derived neurotrophic factor gene
PDC: Phosducin gene
ML: Maximum likelihood
H: Number of haplotypes
Hd: Haplotype diversity
θ_*w*_: Watterson’s theta
Pi: Nucleotide diversity per site
K: Average number of nucleotide differences between sequences
S: Number of segregating sites
ESS: Effective sample sizes
MCC: Maximum clade credibility
BPP: Bayesian phylogenetics and phylogeography
MCMC: Markov chain monte carlo
GMYC: Generalized mixed yule coalescent
bGMYC: Bayesian generalized mixed yule coalescent
RRW: Relaxed random walk
HPD: Highest posterior density
Ma: Mega-annum (i.e., a period of one million years)
SDD: Short-distance dispersals
LDD: Long-distance dispersals
