# Supplementary material for: Biogeographic history and cryptic diversity of saxicolous Tropiduridae lizards endemic to the semiarid Caatinga
Source: BMC Evol Biol. 2015 May 23;15:94. doi: 10.1186/s12862-015-0368-3 (PMC4494643; doi:10.1186/s12862-015-0368-3)
Supplement: Additional file 1: Figure S1. — Mitochondrial DNA (cytb + 16S), BDNF and PDC gene trees for the Tropidurus semitaeniatus species group, as estimated by maximum likelihood (ML). Colors correspond to localities and clades colors in Figure 1 and Figure 2. Figure S2: Tropidurus semitaeniatus species group BDNF and PDC haplotype networks build with HaploViewer. Clade colors are the same as in Figure 1 and 2. Numbers inside the proportionally sized circles represent the number of alleles sharing that particular haplotype. Figure S3: Species tree and divergence times for the Tropidurus semitaeniatus species group and outgroups based on the less inclusive species assignments from the mtDNA haploclades. The maximum clade credibility tree was inferred under a coalescent model based on all three independent loci with *BEAST. Figure S4: Effective population sizes through time based on the Bayesian Skyride as estimated for each of the three main population strains (Clade C) for the T. semitaeniatus complex. Area delimited by the blue line represented the 95%-HPD interval. [file 12862_2015_368_MOESM1_ESM.pdf]

Supporting Info for the paper

Biogeographic history and cryptic diversity of saxicolous Tropiduridae lizards endemic to the semiarid Caatinga

Fernanda P. Werneck, Rafael N. Leite, Silvia R. Geurgas & Miguel T. Rodrigues

Figure S1: Mitochondrial DNA (cytb + 16S), BDNF and Pho gene trees for the *Tropidurus semitaeniatus* species group, as estimated by maximum likelihood (ML). Colors correspond to localities and clades colors in Fig 1 and Fig. 2.

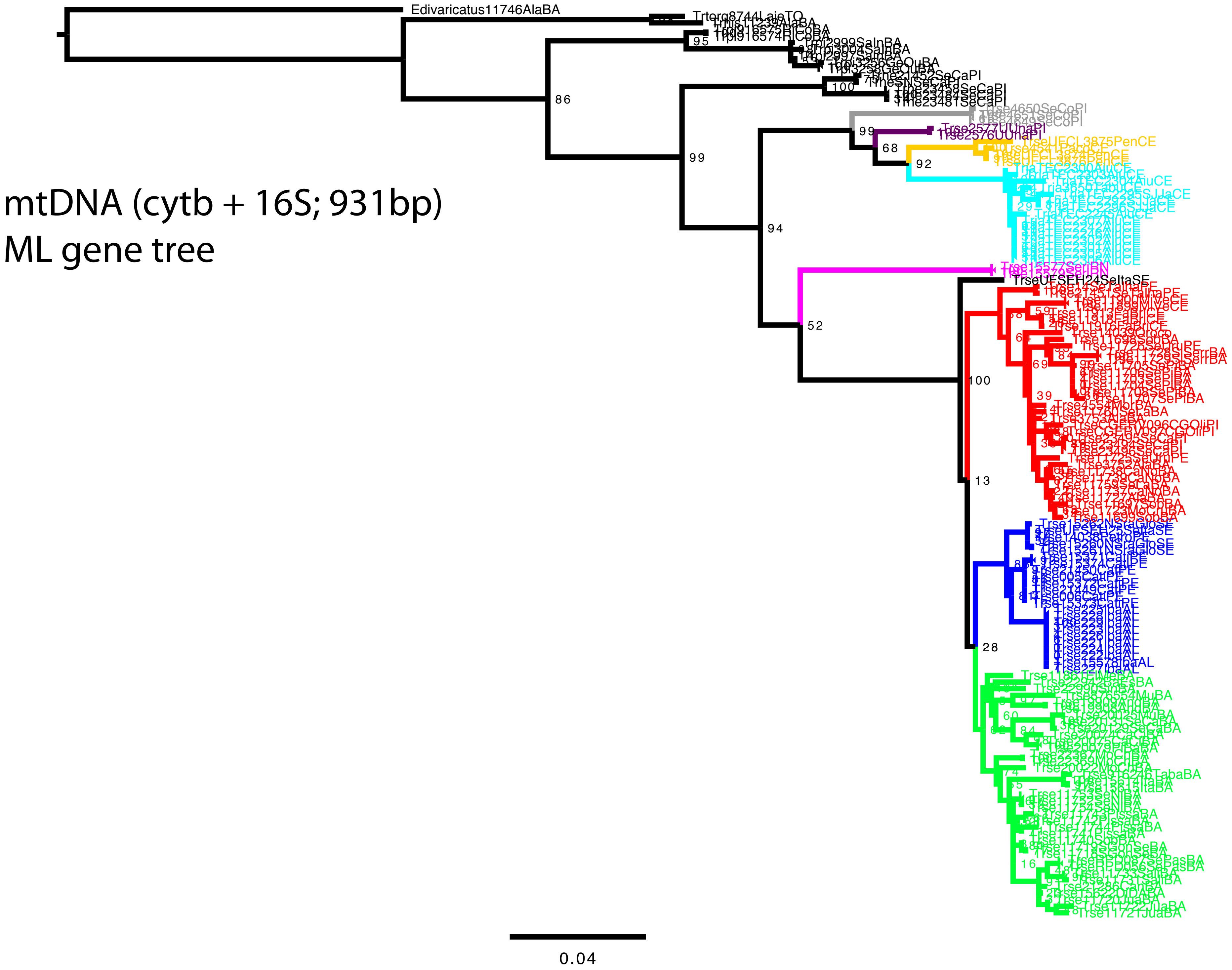
mtDNA (cytb + 16S; 931bp)
ML gene tree
Edivaricatus11746AlaBA
TrseUFSEH24SeltaSE
0.04

BDNF ML gene tree

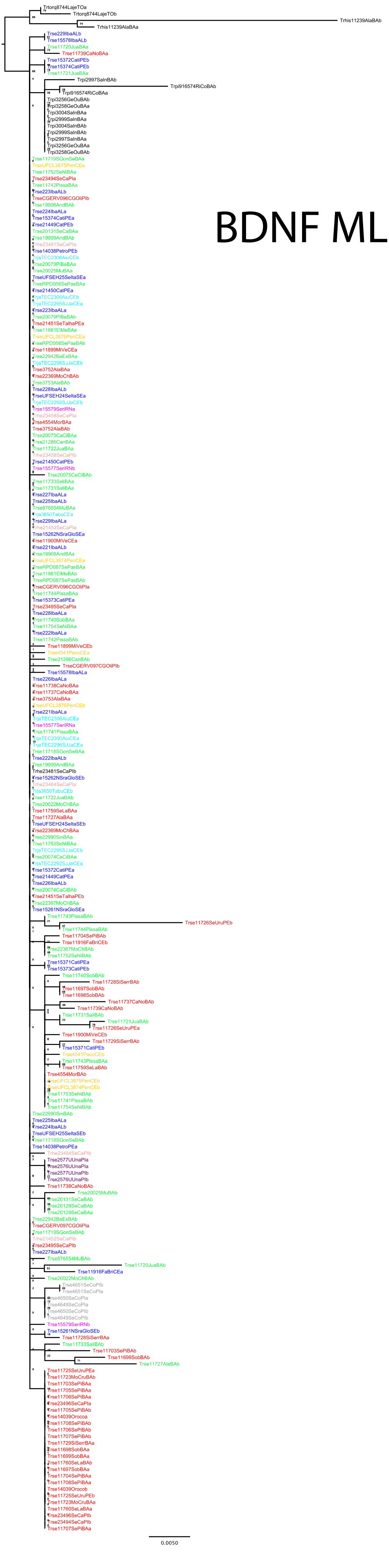

Pho ML gene tree

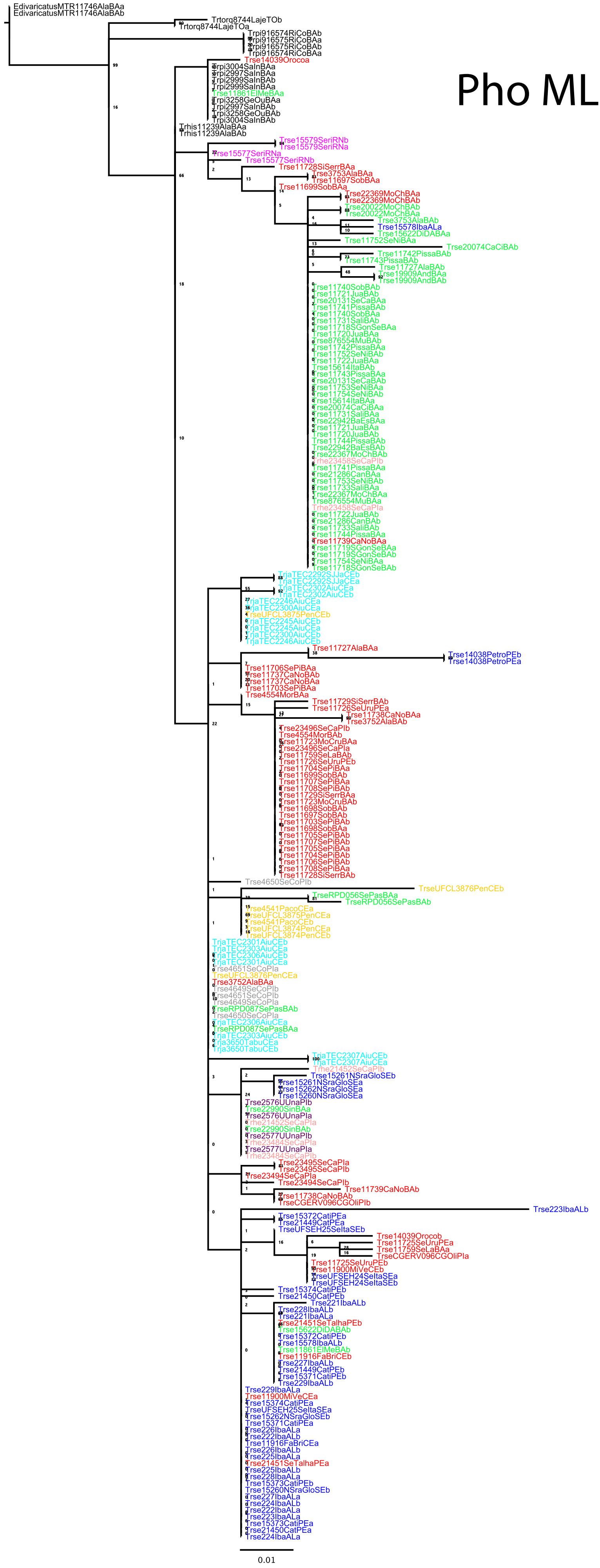

Figure S2: *Tropidurus semitaeniatus* species group BDNF and Pho haplotype networks build with HaploViewer. Clade colors are the same as in Fig. 1 and 2. Numbers inside the proportionally sized circles represent the number of alleles sharing that particular haplotype.

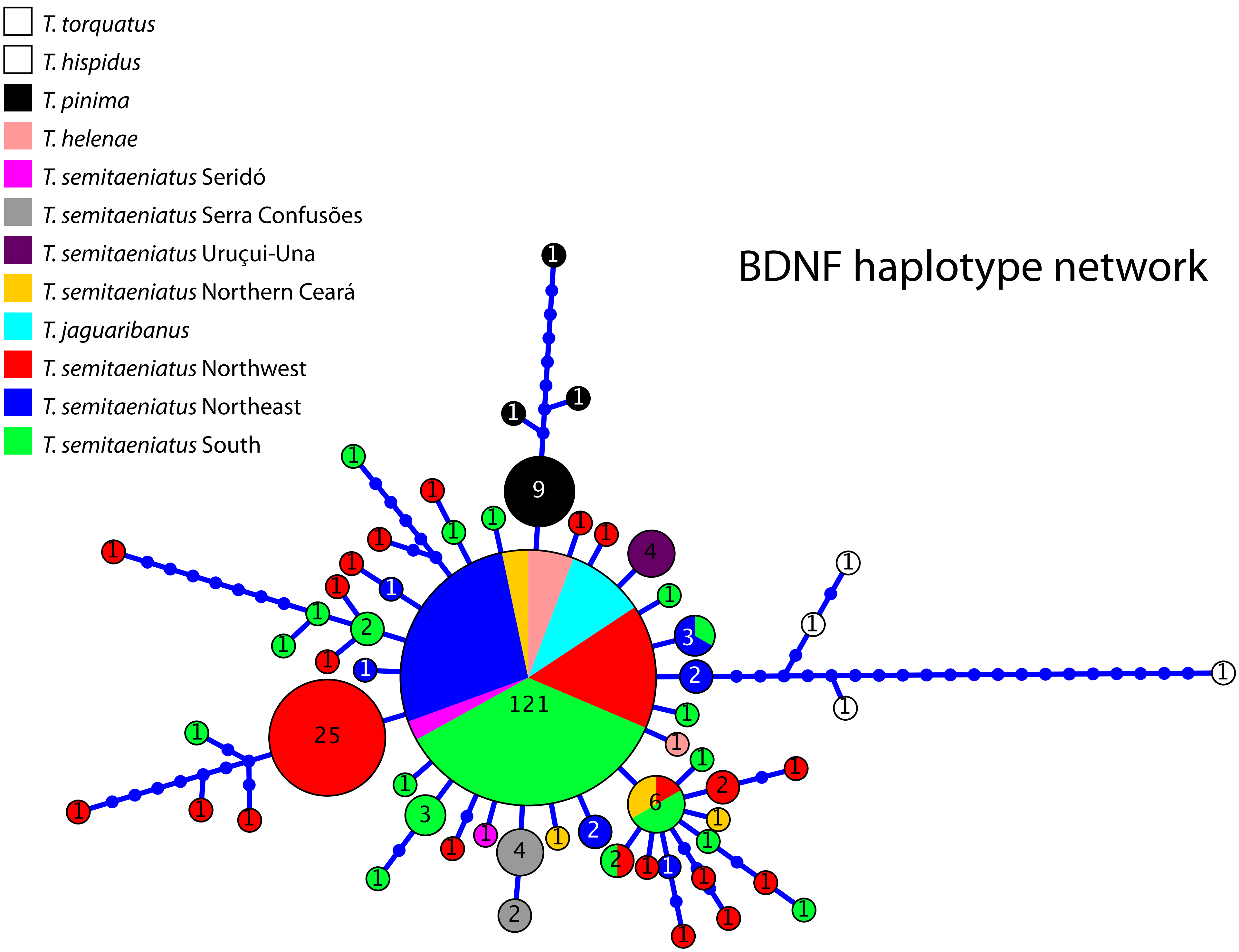
T. torquatus
T. hispidus
T. pinima
T. helenae
T. semitaeniatus Seridó
T. semitaeniatus Serra Confusões
T. semitaeniatus Uruçui-Una
T. semitaeniatus Northern Ceará
T. jaguaribanus
T. semitaeniatus Northwest
T. semitaeniatus Northeast
T. semitaeniatus South
BDNF haplotype network
121
25
9
6
4
4
3
3
2
2
2
2
2

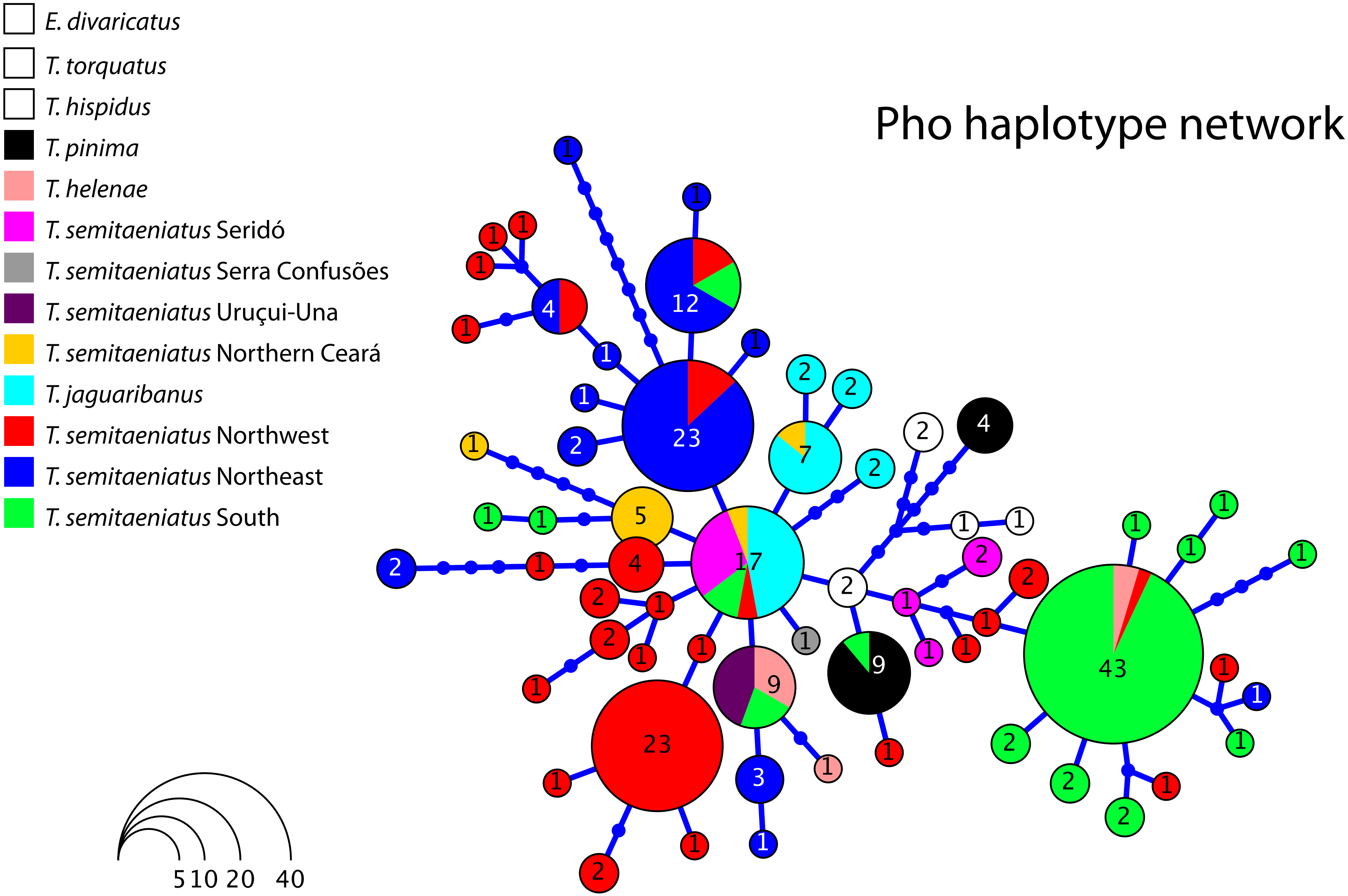

E. divaricatus
T. torquatus
T. hispidus
T. pinima
T. helenae
T. semitaeniatus Seridó
T. semitaeniatus Serra Confusões
T. semitaeniatus Uruçui-Una
T. semitaeniatus Northern Ceará
T. jaguaribanus
T. semitaeniatus Northwest
T. semitaeniatus Northeast
T. semitaeniatus South
Pho haplotype network
5 10 20 40

Figure S3: Species tree and divergence times for the *Tropidurus semitaeniatus* species group and outgroups based on the less inclusive species assignments from the mtDNA haploclades. The maximum clade credibility tree was inferred under a coalescent model based on all three independent loci with *BEAST. Posterior probability (pp) values are indicated by colored circles in the nodes; nodes with no indicative of support have pp < 0.75.

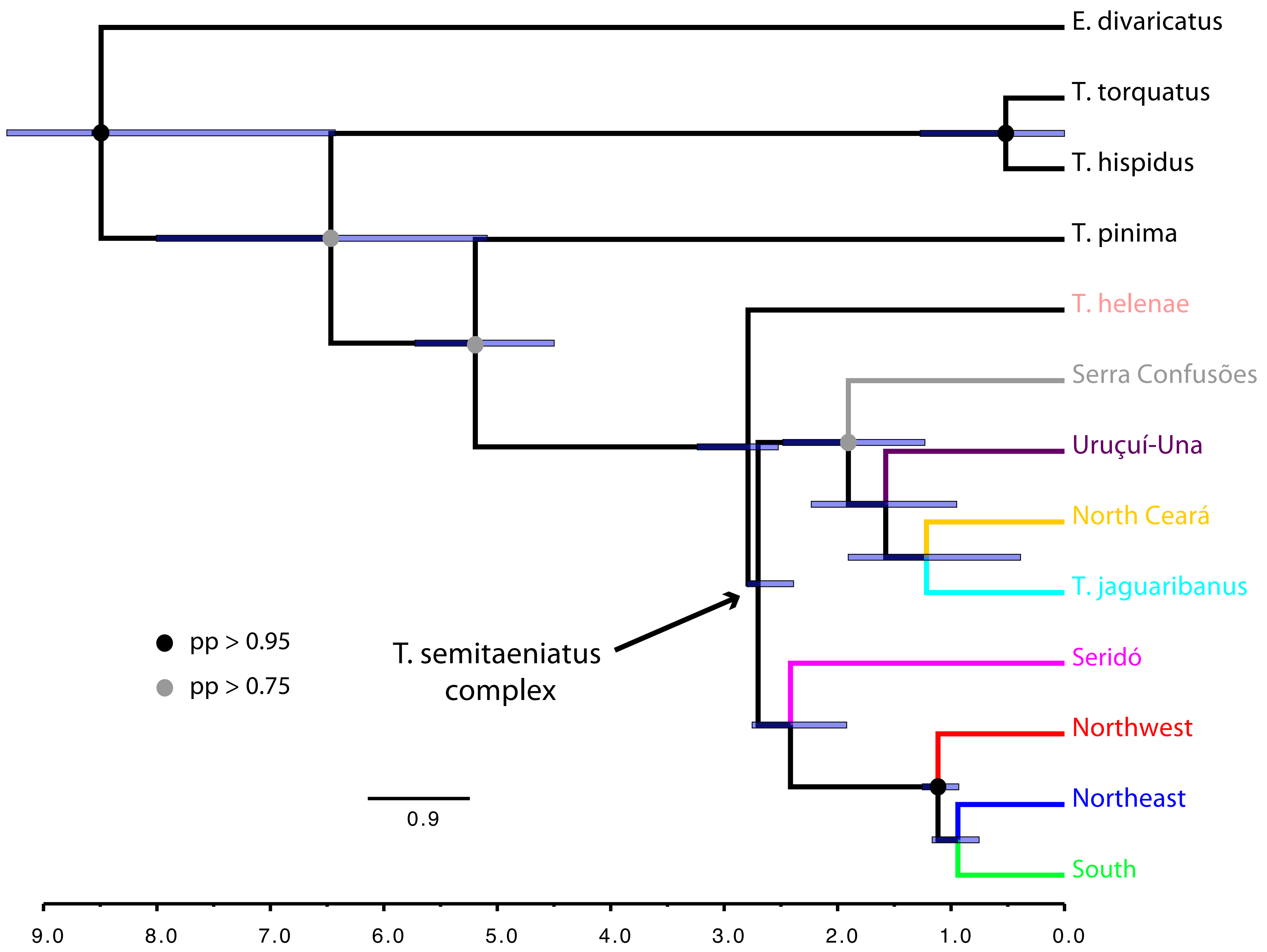
E. divaricatus
T. torquatus
T. hispidus
T. pinima
T. helenae
Serra Confusões
Uruçuí-Una
North Ceará
T. jaguaribanus
Seridó
Northwest
Northeast
South
pp > 0.95
pp > 0.75
T. semitaeniatus
complex
0.9
9.0
8.0
7.0
6.0
5.0
4.0
3.0
2.0
1.0
0.0

Figure S4: Effective population sizes through time based on the Bayesian Skyride as estimated for each of the three main population strains (Clade C) for the T. *semitaeniatus* complex. Area delimited by the blue line represented the 95%-HPD interval.

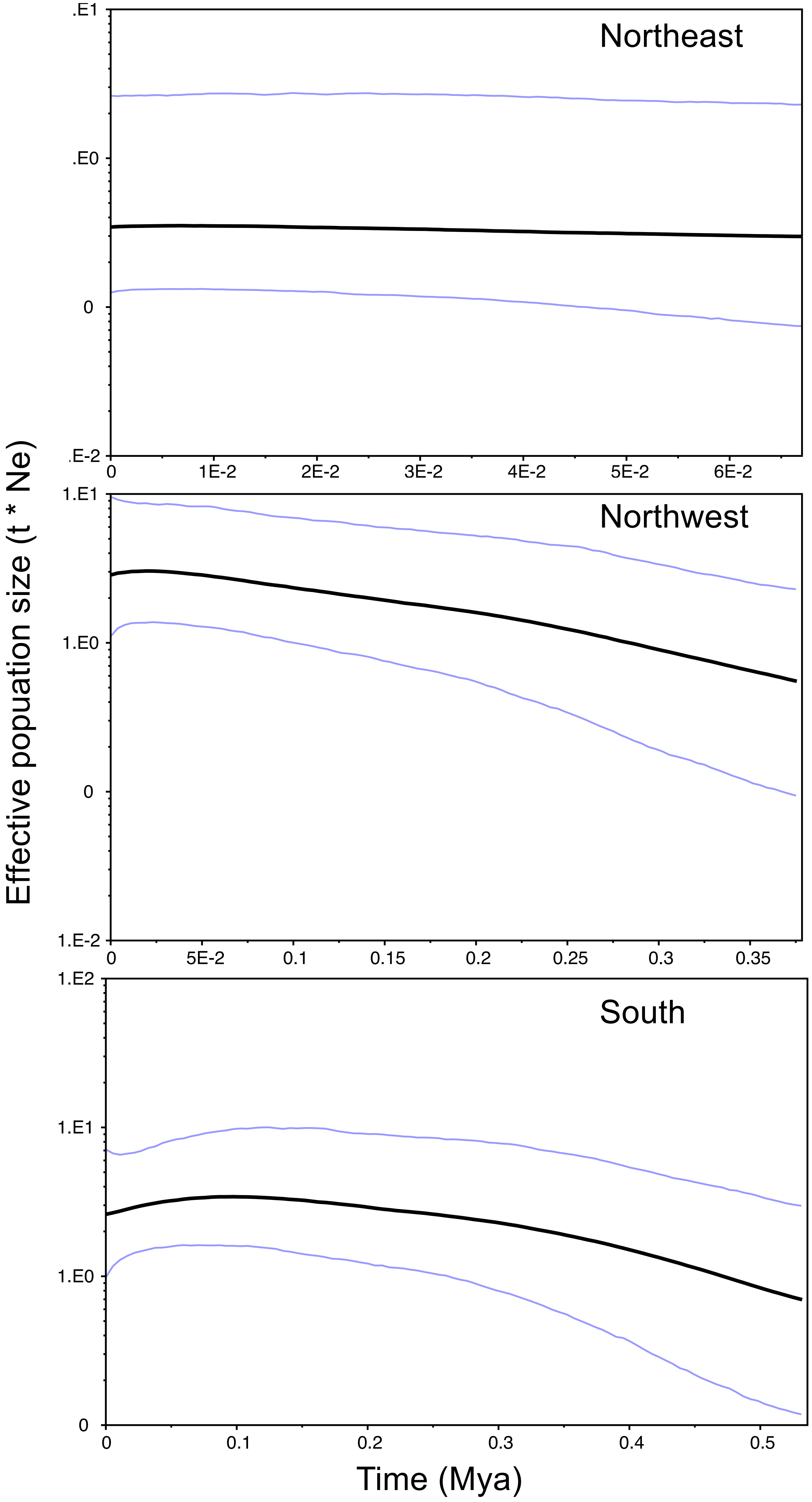
Northeast
.E1
.E0
0
.E-2
0
1E-2
2E-2
3E-2
4E-2
5E-2
6E-2
Northwest
1.E1
1.E0
0
1.E-2
0
5E-2
0.1
0.15
0.2
0.25
0.3
0.35
South
1.E2
1.E1
1.E0
0
0
0.1
0.2
0.3
0.4
0.5
Effective popuation size (t * Ne)
Time (Mya)
